# Disease management in the treatment of patients with chronic heart failure who have universal access to health care: a randomized controlled trial

**DOI:** 10.1186/s12916-017-0855-z

**Published:** 2017-05-01

**Authors:** Ofra Kalter-Leibovici, Dov Freimark, Laurence S. Freedman, Galit Kaufman, Arnona Ziv, Havi Murad, Michal Benderly, Barbara G. Silverman, Nurit Friedman, Tali Cukierman-Yaffe, Elad Asher, Avishay Grupper, Dorit Goldman, Miriam Amitai, Shlomi Matetzky, Mordechai Shani, Haim Silber, for the Israel Heart Failure Disease Management Study (IHF-DMS) investigators, Dan Admon, Miriam Amitai, Michael Arad, Elad Asher, Michal Benderly, Tali Cukierman-Yaffe, Yaakov Dvorkin, Laurence S. Freedman, Dov Freimark, Nurit Friedman, Vered Gercenshtein, Dorit Goldman, Avishay Grupper, Ofra Kalter-Leibovici, Galit Kaufman, Robert Klempner, Lev Lerner, Doron M. Menachemi, Havi Murad, Diab Mutlak, Yael Peled-Potashnik, Shmuel Rispler, Simcha Rosenblatt, Yaron Satanovsky, Mordechai Shani, Ronit Shohat-Zabarski, Haim Silber, Barbara G. Silverman, Edgar Socher, Zvi Vered, Arnona Ziv, Donna R. Zwas

**Affiliations:** 1 Cardiovascular Epidemiology Unit, The Gertner Institute for Epidemiology & Health Policy Research, Chaim Sheba Medical Center, Tel-Hashomer, 5265601 Israel; 2 Sackler Faculty of Medicine, Tel-Aviv University, Tel-Aviv, Israel; 3 Heart Failure Institute, Lev Leviev Heart Center, Chaim Sheba Medical Center, Tel-Hashomer, Israel; 4 Biostatistics Unit, The Gertner Institute for Epidemiology & Health Policy Research, Chaim Sheba Medical Center, Tel-Hashomer, Israel; 5 Maccabi Healthcare Services, Northern District, Israel; 6 Information and Computer Unit, The Gertner Institute for Epidemiology & Health Policy Research, Chaim Sheba Medical Center, Tel-Hashomer, Israel; 7 Israel National Cancer Registry, Israel Center for Disease Control, Gertner Building, Chaim Sheba Medical Center, Tel-Hashomer, Israel; 8 Research and Evaluation Unit, Maccabi Healthcare Services, Tel Aviv, Israel; 9 Endocrinology Institute, Chaim Sheba Medical Center, Tel-Hashomer, Israel; 10 Intensive Cardiac Care Unit, Lev Leviev Heart Center, Chaim Sheba Medical Center, Tel -Hashomer, Israel; 11 Meuhedet Health Services, South District, Israel; 12 Maccabi Healthcare Services, Tel Aviv, Israel; 13 The Medical Research Infrastructure Development and Health Services Fund, Chaim Sheba Medical Center, Tel-Hashomer, Israel

**Keywords:** Disease management, Congestive heart failure, Tele-monitoring, Hospital admissions, Health-related quality of life, Depression, Mortality

## Abstract

**Background:**

The efficacy of disease management programs in improving the outcome of heart failure patients remains uncertain and may vary across health systems. This study explores whether a countrywide disease management program is superior to usual care in reducing adverse health outcomes and improving well-being among community-dwelling adult patients with moderate-to-severe chronic heart failure who have universal access to advanced health-care services and technologies.

**Methods:**

In this multicenter open-label trial, 1,360 patients recruited after hospitalization for heart failure exacerbation (38%) or from the community (62%) were randomly assigned to either disease management or usual care. Disease management, delivered by multi-disciplinary teams, included coordination of care, patient education, monitoring disease symptoms and patient adherence to medication regimen, titration of drug therapy, and home tele-monitoring of body weight, blood pressure and heart rate. Patients assigned to usual care were treated by primary care practitioners and consultant cardiologists.

The primary composite endpoint was the time elapsed till first hospital admission for heart failure exacerbation or death from any cause. Secondary endpoints included the number of all hospital admissions, health-related quality of life and depression during follow-up. Intention-to-treat comparisons between treatments were adjusted for baseline patient data and study center.

**Results:**

During the follow-up, 388 (56.9%) patients assigned to disease management and 387 (57.1%) assigned to usual care had a primary endpoint event. The median (range) time elapsed until the primary endpoint event or end of study was 2.0 (0–5.0) years among patients assigned to disease management, and 1.8 (0–5.0) years among patients assigned to usual care (adjusted hazard ratio, 0.908; 95% confidence interval, 0.788 to 1.047). Hospital admissions were mostly (70%) unrelated to heart failure.

Patients assigned to disease management had a better health-related quality of life and a lower depression score during follow-up.

**Conclusions:**

This comprehensive disease management intervention was not superior to usual care with respect to the primary composite endpoint, but it improved health-related quality of life and depression. A disease-centered approach may not suffice to make a significant impact on hospital admissions and mortality in patients with chronic heart failure who have universal access to health care.

**Clinical trial registration:**

Clinicaltrials.gov identifier: NCT00533013. Trial registration date: 9 August 2007. Initial protocol release date: 20 September 2007.

**Electronic supplementary material:**

The online version of this article (doi:10.1186/s12916-017-0855-z) contains supplementary material, which is available to authorized users.

## Background

Disease management programs for patients with chronic heart failure were introduced in the mid-1990s to improve patient outcomes and reduce health-care costs [1]. Typically, these programs include some or all of the following components: delivery of care by multi-disciplinary teams, patient empowerment and self-care education, coordination of care, reorganization of care delivery systems, use of information systems and reliance on evidence-based practices [2].

Meta-analyses of randomized controlled trials have shown that, compared to usual care, disease management programs reduced hospital admissions and mortality related to heart failure and to all causes among patients with chronic heart failure [3–5]. Nevertheless, some recent large-scale studies in patients with chronic heart failure failed to show any clinical [6–8] or economic [9] benefit of disease management programs over usual care. Some of the conflicting results may be attributed to variations in the types of interventions included in these programs, in patient characteristics and in the quality of care given to patients assigned to the control group [10]. Usual care may be more effective in achieving a good outcome among heart failure patients in health systems that provide universal access to health care.

In Israel, the National Health Insurance Law enables universal access to primary, secondary and tertiary care and advanced health technologies, which are delivered by four health maintenance organizations.

We aimed to evaluate the long-term effect of a countrywide comprehensive disease management program among patients with chronic heart failure, insured by Maccabi Health Services, the second largest health maintenance organization in Israel.

## Methods

### Study design

In this multicenter open-label randomized trial, we compared the efficacy of a disease management program versus usual care among ambulatory adult patients (age ≥18 years) with moderate-to-severe chronic heart failure [New York Heart Association (NYHA) functional classes II to IV [11]]. Patients were referred within 2 months after hospital admission for heart failure exacerbation or from the community, by nurse supervisors at the public hospitals or by primary care practitioners and consultant cardiologists in the community, respectively. Screening for study eligibility was carried out in ten designated community heart failure centers. Diagnosis of heart failure was based on typical signs and symptoms and objective echocardiographic evidence of functional or structural abnormality of the heart at rest [12]. Patients with severe comorbidity, functional or cognitive impairment, or substance abuse were excluded (detailed information on eligibility criteria is available in the study protocol, see Additional file 1).

After completing eligibility and baseline assessments and providing signed informed consent, patients were randomly assigned to either disease management or usual care, using a computerized randomization program with a permuted-block design linked to the patients’ electronic medical record. Randomization was in a 1:1 ratio and stratified by heart failure center. The study safety committee reviewed the randomization process and found no evidence of violation or tampering with the randomization protocol.

The study was approved by the Maccabi Health Services and the Sheba Medical Center research ethics committees.

### Interventions

Patients enrolled in disease management were assigned to nurses who maintained regular remote contact with them between their scheduled visits to the heart failure centers, either through telephone calls or computer video sessions. During these sessions, the nurses provided comprehensive care and support to the patients and their caregivers, including self-care education, monitoring disease signs and symptoms, titration of heart failure medications following designated protocols, monitoring adherence to the medical therapy and its side effects, coordination of care vis-à-vis other caregivers and counselling in the event of an acute change in health status. All disease management activities were recorded in the patients’ electronic medical records and supervised by the program director. The initial frequency of remote contacts with patients assigned to disease management was once a week and was further modified according to needs. Patients monitored their body weight, blood pressure and pulse rate daily, after a night's sleep and voiding, using home tele-monitoring equipment (Medic4All®). The tele-monitoring records were transmitted to the patients’ electronic medical records. Automatic alerts were generated and presented to the nurses if the signals were out of the patient-specific preset range.

The frequency of follow-up visits at the heart failure centers was determined according to the patients’ needs, but was not less than once every 6 months. During these visits, the patients were evaluated by cardiologists and nurses, and their treatment plan was modified accordingly. Counselling by dietitians and social workers at the heart failure center was provided when needed.

Patients assigned to usual care were referred to their primary care practitioners, after clinical assessment and provision of a treatment plan by cardiologists at the heart failure center. In general, ambulatory heart failure patients insured with Maccabi Health Services are cared for by primary care practitioners and consultant cardiologists. The frequency of visits to the cardiologists was determined according to the patients’ needs. In addition, patients assigned to usual care were evaluated every 6 months by cardiologists at the heart failure center. These patients did not receive any intervention from the nurses at the call center, or from dietitians and social workers at the heart failure centers (detailed information is also available in the study protocol, see Additional file 1).

### Study assessment and endpoints

All patients were evaluated at the heart failure centers at baseline and at 6-month intervals thereafter. These assessments included obtaining patient history and patient-completed questionnaires, a 6-minute walk test and NYHA classification. From 2009 onwards, assessments included also point-of-care brain natriuretic peptide (BNP) testing (using Triage® BNP Test and Triage® MeterPro, Alere, Switzerland). Except for the baseline evaluation, assessors were not blinded to the patients’ assigned intervention.

The primary composite endpoint was the time to first hospital admission for heart failure or to death from any cause. Secondary endpoints included the individual components of the primary composite endpoint; the total number of hospital admissions and in-hospital days for heart failure and for all causes; follow-up assessments of a 6-minute walk test, NYHA classification, health-related quality of life assessed with the 36-item short-form questionnaire (SF-36) [13], depression symptoms assessed with the nine-item patient health depression scale (PHQ-9) [14], BNP levels (in a subset of patients), and purchases of recommended medications for patients with chronic heart failure [i.e. angiotensin-converting enzyme inhibitors (ACE-Is), angiotensin receptor blockers (ARBs) and beta adrenergic receptor blocking agents [12]].

Information on hospital admissions and deaths during follow-up was obtained. Discharge summaries, available for 5,748 (99.7%) of 5,766 hospital admissions, were adjudicated by two independent investigators blinded to the patients’ assigned treatment, and were classified as either related to heart failure (i.e. decompensated heart failure, complications of heart failure or heart failure treatment) or other causes. Disagreement between the assessors occurred for 581 (10.1%) of the hospital discharge summaries, and a third assessment was provided by a third blinded investigator, with the final classification following the majority opinion.

### Safety committee

A safety committee, comprising a statistician, cardiologist and epidemiologist, monitored the cumulative event rates of hospital admissions and deaths from all causes among the study participants every 6 months. The committee members were not blinded to the participants’ assigned intervention. The committee discussions were not disclosed to the investigators during the trial. Guided by a predefined level of statistical significance [15], the mandate of the committee was to provide warning in the event of an excess number of deaths or hospital admissions from all causes in the disease management arm. In practice, no such excess was found and the study continued to its planned conclusion.

### Sample size consideration

The planned sample size of 1,200 patients, 600 in each treatment arm, was based on the assumption that 50% of the participants assigned to the usual care arm would be admitted to hospital for heart failure or die during the first 12 months and that the odds ratio (OR) for this primary endpoint was 0.67 for disease management versus usual care. The assumptions were based on a published meta-analysis of previous disease management trials among heart failure patients [3]. This sample size provided 90% statistical power, using a two-sided 0.05 significance level. The final study sample included 1,360 patients, to account for drop-outs and the time needed for organizational learning and adaptation.

### Statistical analysis

The statistical analysis was performed according to a pre-specified plan. Unadjusted comparisons between the two treatment groups with respect to the primary composite endpoint (time to first hospital admission due to heart failure or death from any cause) and its two individual components were made using the log-rank statistic.

The Cox proportional hazards model was used to compare the two treatment groups with respect to the primary composite endpoint and its components, adjusted for baseline characteristics (sex, age, heart failure center, 6-minute walk test and NYHA classification). In supportive analyses, adjustment was also made for other baseline variables that were significantly associated with hospital readmission due to heart failure or death from any cause (including source of recruitment, the main underlying cause of heart failure, body mass index, renal failure and hemoglobin level). The median time to first hospital admission for heart failure and its 95% confidence interval were estimated in the two treatment groups, standardized to the most common patient baseline profile using an inverse transformation of the predicted survival curve at 50%. Predefined interactions between the treatment group and baseline variables were also tested. The proportional hazards assumption for the treatment group was tested in all of these models.

To compare hospital admission data recorded every 6 months in the two treatment groups, non-linear mixed models were used with a random intercept for each subject, allowing for correlation between repeated measurements of the same subject. Due to the over-dispersion commonly seen in hospital admission data [16], a negative binomial model was used. Using the NLMIXED procedure (SAS version 9.4), this method was implemented for the following four secondary outcomes: number of hospital admissions and days of hospitalization, for heart failure and all causes. These models were adjusted for baseline characteristics and time since randomization in intervals of 6 months, and effectively dealt with the varying length of follow-up among individuals due to censoring. The coefficient of the treatment group variable represented the log incidence rate ratio for 6 months. Absolute differences between treatment groups in the number of hospital admissions and in-hospital days over the first 2 years were calculated from the estimated incidence rate ratio and the mean outcome in the usual care group.

Other secondary endpoints were dichotomously categorized to represent successful attainment (or not) of a minimal clinically important difference from baseline, as follows: physical component summary and mental component summary scores of SF-36 (≥2.5 points increase [17]); NYHA classification (≥1 class decrease [18]); 6-minute walk test (≥50 m increase [19]), and moderate-to-severe depression symptoms at follow-up (PHQ-9 score ≥10 [14]). The treatment OR of success was estimated using a logistic model within the same mixed-model framework described above. Interactions between the treatment group and baseline value of the endpoint were tested.

Using the Maccabi Health Services database on drug purchases, adherence to medical therapy was defined as the number of days in a 6-month period covered by therapy with ACE-I/ARBs and beta adrenergic receptor blocking agents according to the defined daily dose [20], classified into five categories (the first category representing no treatment and the second to the fifth categories representing defined daily dose quartiles for patients taking the medication). The treatment OR of attaining a higher level of coverage by drug therapy was estimated using an ordered logistic regression model within the mixed-model framework. The assumption of proportional odds for each cut-point of the adherence variables was tested.

All analyses were carried out according to the intention-to-treat principle, at a critical two-sided significance level of 0.05.

## Results

Participants were recruited between August 2007 and June 2011, and followed until death or end of study (July 2012). The median time of follow-up was 2.7 years (range 0–5).

**Fig. 1:**
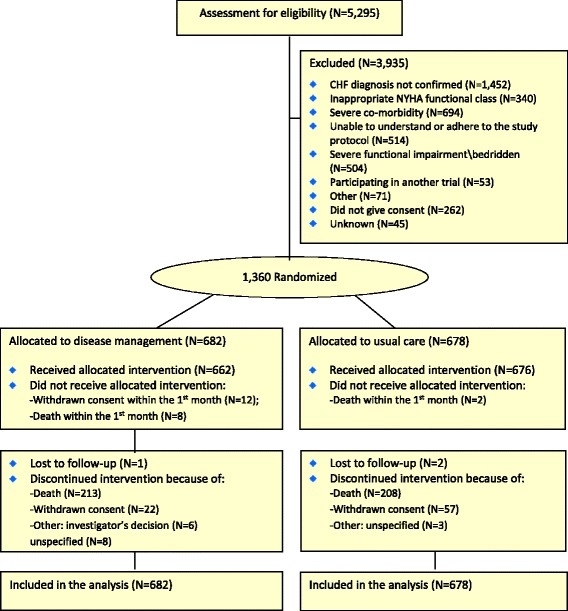
Screening, randomization and completion of follow-up. *CHF* congestive heart failure, *NYHA* New York Heart Association

A total of 5,295 patients were screened for eligibility, of whom 3,673 were found non-eligible, 262 declined participation, and 1,360 were randomly assigned either to usual care (678) or to disease management (682). Altogether, 22 participants did not receive the allocated intervention; 12 because of withdrawn consent and because ten participants died within 1 month after randomization. Three participants were lost to follow-up and 96 discontinued the intervention (Fig. 1). All randomized patients were included in the analyses.

The mean age (standard deviation) of participants was 70.7 (11.3) years, and 987 (72.5%) were men. Most were NYHA functional class III (79%) and 82% had reduced left ventricular ejection fraction (<50%). Nine NYHA class I patients were recruited by mistake (five assigned to disease management and four to usual care). At enrolment, a large proportion of participants were taking medication recommended for treating chronic heart failure. Compared to patients assigned to usual care, those assigned to disease management were less likely to be male, were more likely to be NYHA functional class IV and performed slightly worse on a 6-minute walk test. Although statistically significant, the absolute differences between the two study groups for these variables were small. Otherwise, the baseline characteristics of the patients were similar across the two groups (Table 1).

**Table 1:** Baseline characteristics of the study participants

	Disease management (*N* = 682)		Usual care (*N* = 678)		*P*
	Mean	(s.d.)	Mean	(s.d.)	
Age (years)	70.8	(11.6)	70.7	(11.0)	0.49
	*n*	(%)	*n*	(%)	
Male	473	(69.3)	513	(75.7)	0.009
Source of recruitment					0.33
Recent hospital admission for heart failure	270	(39.6)	251	(37.0)	
Community	412	(60.4)	427	(63.0)	
Main cause of heart failure: ischemic heart disease	477	(69.9)	489	(72.1)	0.37
Left ventricular ejection fraction					0.085
Preserved (>50%)	136	(20.2)	111	(16.6)	
Reduced (<50%)	537	(79.8)	559	(83.4)	
NYHA functional class					0.005^a^
I	5	(0.7)	4	(0.6)	
II	81	(11.9)	116	(17.1)	
III	543	(79.9)	528	(78.0)	
IV	51	(7.5)	29	(4.3)	
	Median	IQR	Median	IQR	
6-minute walk test (m)	165	(80, 274)	200	(90, 306)	0.002
Brain natriuretic peptide (pg/mL)^b^	323	(145, 768)	295	(148, 547)	0.066
	*n*	(%)	*n*	(%)	
Chronic atrial fibrillation^c^	156	(23.4)	177	(26.9)	0.14
	*n*	(%)	*n*	(%)	
Treatment					
Angiotensin converting enzyme inhibitors/ Angiotensin receptor blockers	570	(83.6)	567	(83.6)	0.98
Beta adrenergic receptor blockers	567	(83.1)	569	(83.9)	0.70
Aldosterone antagonists	256	(37.5)	263	(38.8)	0.63
Diuretics	629	(92.2)	613	(90.4)	0.23
Platelet anti-aggregants	486	(71.3)	479	(70.6)	0.80
Statins	527	(77.3)	534	(78.8)	0.51
Pacemaker	96	(14.1)	119	(17.5)	0.079
Implantable cardioverter defibrillator	115	(16.9)	113	(16.7)	0.92
	Median	IQR	Median	IQR	
Health-related quality of life score (SF-36)					
Physical	38	(27, 53)	41	(30, 54)	0.011
Mental	46	(37, 58)	48	(38, 58)	0.34
Depression symptoms score (PHQ-9)	9	(4, 14)	8	(4, 13)	0.28
	*n*	(%)	*n*	(%)	
History of cardiovascular disease					
Myocardial infarction	418	(61.6)	441	(65.0)	0.18
Percutaneous coronary intervention	280	(41.2)	291	(42.9)	0.53
Coronary artery bypass grafting	219	(32.2)	236	(34.8)	0.32
Stroke/transient ischemic attack	103	(15.2)	106	(15.6)	0.81
Peripheral vascular disease	128	(18.8)	126	(18.6)	0.90
	*n*	(%)	*n*	(%)	
Other comorbidity					
Diabetes mellitus	354	(52.2)	339	(50.0)	0.41
Hypertension	504	(74.2)	506	(74.7)	0.83
Dyslipidemia	518	(76.3)	523	(77.1)	0.71
Chronic obstructive pulmonary disease	122	(18.0)	105	(15.5)	0.22
Renal failure^d^	377	(56.5)	378	(56.8)	0.93
	Mean	(s.d.)	Mean	(s.d.)	
Hemoglobin (gr/dL)	12.5	(2.0)	12.8	(1.8)	0.08
Body mass index (kg/m^2^)	30.0	(5.9)	29.8	(5.5)	0.36

*NYHA* New York Heart Association, *IQR*, interquartile range, *PHQ-9* nine-item patient health depression scale, *s.d.* standard deviation, *SF-36* 36-item short form

^a^Fisher’s exact test

^b^Baseline brain natriuretic peptide data were available for 377 patients assigned to disease management and 373 patients assigned to usual care

^c^Information on presence of atrial fibrillation was missing for 34 patients

^d^Estimated glomerular filtration rate < 60 mL/min/1.73 m^2^ signifies renal failure

### Endpoints

During 3,421 patient-years, there were 5,766 hospital admissions for 1,184 participants; 1,707 hospital admissions for 628 participants were for heart failure, and 450 participants died (Table 2).

**Table 2:** Total and per study group visits to a cardiologist, hospital admissions and deaths during follow-up

	Total (*N* = 1,360)		Disease management (*N* = 682)		Usual care (*N* = 678)	
	Mean	(s.d.)	Mean	(s.d.)	Mean	(s.d.)
Length of follow-up (years)	2.67	(1.22)	2.68	(1.21)	2.67	(1.22)
	*n*	(%)	*n*	(%)	*n*	(%)
Primary endpoint events^a^	775	(57.0)	388	(56.9)	387	(57.1)
Deaths from all causes	450	(33.1)	232	(34.0)	218	(32.1)
Hospital admissions for all causes	(*N* = 1,184)		(*N* = 587)		(*N* = 597)	
	*n*		*n*		*n*	
Total number of admissions	5,766		2,913		2,853	
Total number of hospital days	30,540		15,296		15,244	
Hospital admissions for heart failure	(*N* = 628)		(*N* = 302)		(*N* = 326)	
Total number of hospital admissions	1,707		857		850	
Total number of hospital days	10,235		5,029		5,206	
	Mean	(s.d.)	Mean	(s.d.)	Mean	(s.d.)
Visits to a cardiologist during follow-up (number/year)	6.8	(5.6)	7.9	(6.3)	5.6	(4.5)
Visits to a primary practitioner during follow-up (number/year)	21.5	(13.7)	22.2	(14.4)	20.9	(12.9)

*s.d.* standard deviation

^a^The primary endpoint was defined as the first hospital admission for heart failure or death from any cause

### Primary endpoint and its components

**Fig. 2:**
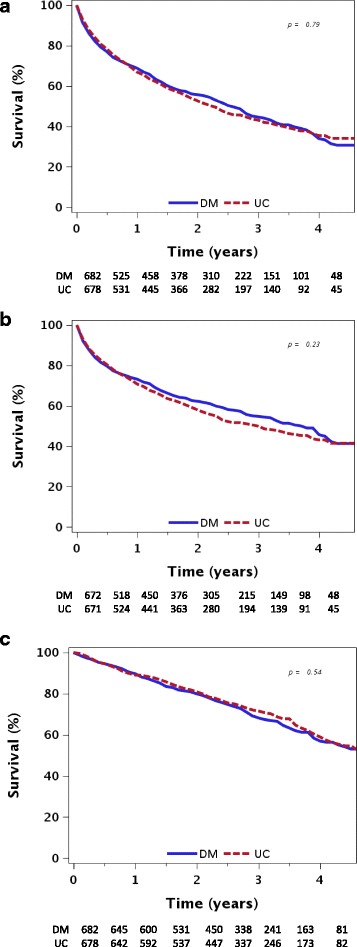
**a** First hospital admission for heart failure or death from all causes by study group. **b** First hospital admission for heart failure by study group. **c** Death from all causes by study group. The *p* value refers to a comparison between the two study groups using a log-rank test. *DM* Disease management, *UC* Usual care

The primary composite endpoint, first hospital admission for heart failure or death from any cause, occurred in 388 (56.9%) patients assigned to disease management, and in 387 (57.1%) patients assigned to usual care. The median (range) time to a primary endpoint event or end of follow-up was 2.0 (0–5.0) years among patients assigned to disease management, and 1.8 (0–5.0) years among patients assigned to usual care [hazard ratio (HR), 0.981; 95% confidence interval (CI), 0.852 to 1.129] (Fig. 2a). Further adjustment for age, sex, heart failure center, and baseline NYHA classification and 6-minute walk test did not show a statistically significant advantage of disease management over usual care (adjusted HR, 0.908; 95% CI, 0.788 to 1.047) (Table 1).

Disease management delayed the time to first hospital admission for heart failure (adjusted HR, 0.832; 95% CI, 0.708 to 0.977). The median (95% CI) time to first hospital admission for heart failure, estimated for a participant with a typical profile (72.5-year-old male, NYHA functional class III, recruited from the community with ischemic heart failure, renal failure and median values of hemoglobin and 6-minute walking distance) was 3.0 years (2.3 to 3.8) if assigned to disease management versus 2.2 years (1.8 to 2.8) if assigned to usual care.

There two treatment groups did not differ by mortality from all causes (adjusted HR, 0.997; 95% CI, 0.820 to 1.213) (Table 3 and Fig. 2c).

**Table 3:** The effect of the intervention (disease management versus usual care) on the primary endpoint and its components

Endpoint	Crude hazard ratio (95% confidence interval)	Adjusted hazard ratio^a^ (95% confidence interval) (model 1)	Adjusted hazard ratio^a^ (95% confidence interval) (models 2, 3)
Time to first hospital admission for heart failure or death from all causes (primary composite outcome)	0.981 (0.852 to 1.129)	0.908 (0.788 to 1.047)	–
Time to death from all causes	1.060 (0.881 to 1.275)	0.982 (0.814 to 1.185)	0.997 (0.820 to 1.213) (Model 2)
Time to first hospital admission for heart failure	0.909 (0.777 to 1.063)	0.846 (0.722 to 0.991)	0.832 (0.708 to 0.977) (Model 3)

*NYHA* New York Heart Association

^a^Cox proportional hazard models were adjusted for

Model 1: Sex, study center, baseline age, NYHA classification and 6-minute walk test

Model 2: All covariates included in model 1 plus source of recruitment, body mass index, renal failure and hemoglobin

Model 3: All covariates included in model 1 plus source of recruitment, main underlying cause of heart failure, baseline renal failure and hemoglobin

Renal failure was defined as an estimated glomerular filtration rate less than 60 mL/min/1.73 m^2^

**Fig. 3:**
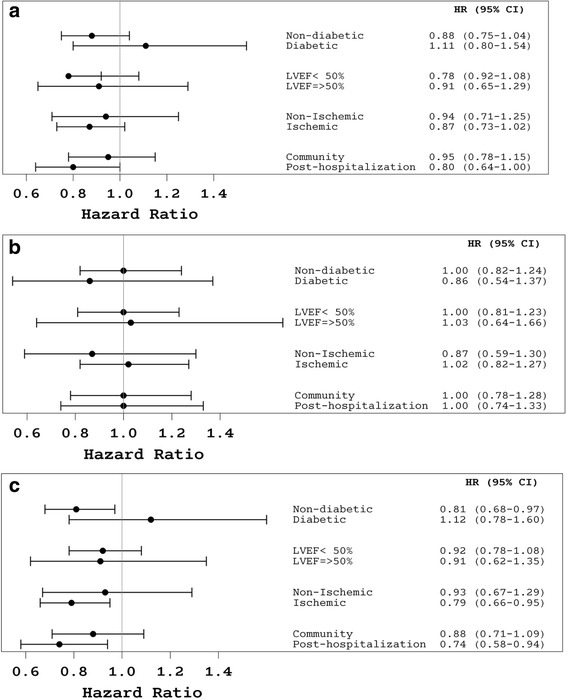
**a** Effect of disease management on the composite outcome (first hospital admission for heart failure or death) by subgroups of patients. **b** Effect of disease management on all-cause mortality by subgroups of patients. **c** Effect of disease management on first hospital admission for heart failure by subgroups of patients. Information on the effect of disease management in subgroups of patients was derived from Cox proportional hazard models, adjusted for age, sex, heart failure center and baseline values of NYHA functional class and 6-minute walk test. *NYHA* New York Heart Association

None of the pre-specified interactions tested were found statistically significant, with respect to the intervention effect on the primary endpoint or its components. Nevertheless, among patients recruited after recent hospital admission for heart failure or with ischemic heart failure, the disease management intervention was associated with a lower hazard for first hospital admission for heart failure compared to usual care [corresponding adjusted HRs (95% CIs): 0.74 (0.58 to 0.94) and 0.79 (0.66 to 0.95); see more detailed information for pre-specified patient subgroups in Fig. 3].

### Other secondary endpoints

The total number of hospital admissions and in-hospital days for heart failure and for all causes during follow-up did not significantly differ between the two study groups. On average, the number of in-hospital days for heart failure among patients assigned to disease management was 28% lower than among patients assigned to usual care [adjusted rate ratio (RR): 0.720; 95% CI, 0.508 to 1.021] (Table 4). Compared to a patient assigned to usual care, on average, a patient assigned to disease management stayed about 1.6 fewer days in the hospital for heart failure during the first 2 years of follow-up (95% CI, −2.9 to +0.1 days). Similarly, the average number of heart failure hospital admissions was 18% lower among patients assigned to disease management than among patients assigned to usual care (adjusted RR: 0.816; 95% CI, 0.665 to 1.001) (Table 4). Compared to a patient assigned to usual care, on average, a patient assigned to disease management had about 0.2 fewer heart failure hospital admissions during the first 2 years of follow-up (95% CI, −0.3 to 0.0; *p* = 0.051).

**Table 4:** The effect of the intervention (disease management versus usual care) on hospital admission endpoints

Endpoint	Crude incidence rate ratio (95% confidence interval)	Adjusted incidence rate ratio^a^ (95% confidence interval)
Number of hospital admissions for heart failure	0.894 (0.723 to 1.106)	0.816 (0.665 to 1.001)
Total number of in-hospital days for heart failure	0.846 (0.596 to 1.200)	0.720 (0.508 to 1.021)
Number of hospital admissions for all causes	0.973 (0.870 to 1.089)	0.935 (0.840 to 1.040)
Number of total in-hospital days for all causes	0.943 (0.793 to 1.120)	0.886 (0.749 to 1.048)

*NYHA* New York Heart Association

^a^Incidence rate ratios between the expected mean number of hospital admissions and in-hospital days among patients assigned to disease management and patients assigned to usual care were derived from negative binomial non-linear mixed models, adjusted for study center; source of recruitment; year at recruitment; study period; sex; baseline age, NYHA classification and 6-minute walk test

There were no statistically significant differences between the two treatment groups with respect to the total number of in-hospital days (adjusted RR: 0.886; 95% CI, 0.749 to 1.048), and the total number of hospital admissions for all causes (adjusted RR: 0.935; 95% CI, 0.840 to 1.040).

Patients assigned to disease management were less likely to experience moderate-to-severe depression symptoms and more likely to experience clinically important improvements in health-related quality of life than those assigned to usual care. They were also more likely to improve their NYHA functional class. There were no significant differences between the treatment groups with respect to a clinically important change in the 6-minute walk test (Table 5).

**Table 5:** The effect of the intervention (disease management versus usual care) on other secondary outcome and process endpoints, adjusted for baseline characteristics

Endpoint	Adjusted odds ratio (95% confidence interval)^a^
Depression symptoms^b^	0.688 (0.528 to 0.897)
Health-related quality of life^c^	
Physical component summary	1.531 (1.165 to 2.011)
Mental component summary	1.571 (1.253 to 1.971)
NYHA functional class^d^	1.477 (1.107 to 1.972)
6-minute walk test^e^	1.334 (0.929 to 1.915)
Adherence to treatment with angiotensin converting enzyme inhibitors or angiotensin receptor blocking agents^f^	1.010 (0.810 to 1.261)
Adherence to treatment with beta adrenergic receptor blocking agents^f^	0.933 (0.767 to 1.134)

*NYHA* New York Heart Association

^a^Odds ratios derived from non-linear mixed models, adjusted for study center; year at recruitment; study period; sex; baseline value of the endpoint variable, age, NYHA function classification, and 6-minute walk test

^b^Measured with the nine-item patient health depression scale (PHQ-9). PHQ-9 score was classified as <10 (no or mild depression) and ≥10 (moderate-to-severe depression) [14]. Odds ratios were calculated for PHQ-9 score ≥10 during follow-up, tested in the ordered logistic non-linear mixed model

^c^Measured with 36-item short-form questionnaire (SF-36) [13]. Odds ratios were calculated for a ≥2.5 point increase in physical component summary and mental component summary scores

^d^Odds ratios were calculated for a 1-class decrease in NYHA functional classification

^e^Odds ratios were calculated for a ≥50 m increase in 6-minute walking distance

^f^Adherence was defined as the number of treatment days covered according to defined daily dose and measured at 6-month intervals. The categories for number of days covered were: 0, 1–90, 91–150, 151–196 and >196. Odds ratios were calculated for attaining a category for number of days covered during follow-up, tested in the ordered logistic non-linear mixed model

There were also no significant differences between the treatment groups with respect to the level of coverage for therapy by ACE-Is/ARBs or beta adrenergic receptor blocking agents during follow-up (Table 5).

### Process variables

The mean (standard deviation) number of remote contacts with a nurse for patients assigned to disease management was 27.3 (17.8) during the first year after enrolment and 18.8 (13.7) per year during the total stay in the program.

The mean (standard deviation) number of annual visits to a cardiologist during the total follow-up period was 7.9 (6.3) for patients assigned to disease management and 5.6 (4.5) for patients assigned to usual care (*p* < 0.001). There was no significant difference in the mean number of visits to a primary practitioner between patients assigned to disease management and those assigned to usual care [mean (standard deviation) number of visits per year; 22.2 (14.4) and 20.9 (12.9), respectively; *p* = 0.141] (Table 2).

## Discussion

In this trial, treatment with disease management was not superior to usual care with respect to the primary outcome, i.e. time to first hospital admission for heart failure or death from any cause. The study intervention prolonged the time to first hospital admission for heart failure, especially among patients enrolled after recent hospitalization for heart failure or with ischemic heart failure. There was also a trend, although not statistically significant, towards a reduction in the number of hospital admissions and in-hospital days for heart failure. The intervention was also effective in achieving clinically important improvements in health-related quality of life and it reduced the likelihood of depression.

The disease management intervention tested in this study was comprehensive and included delivery of care by multi-disciplinary teams within designated heart failure centers and a central call center, home tele-monitoring, employment of information technology to promote sharing of patient information among all caregivers and utilization of standardized care processes. Nevertheless, the intervention did not conclusively reduce the number of hospitalizations due to all causes, nor did it reduce mortality. In this respect, our study confirms the results of recent studies showing little or no effect of disease management in reducing recurrent hospital admissions or mortality in ambulatory patients with chronic heart failure [6–8]. Much of the marginal effect of disease management interventions tested in controlled trials depends upon the type of care delivered to patients assigned to the control group. In Israel, there is universal access to primary, secondary and tertiary health-care services and advanced health-care technologies. This was reflected by the high proportion of patients receiving evidence-based drug therapy upon recruitment and the small difference between the treatment groups with respect to the number of contacts with cardiologists during follow-up.

The intervention tested in this study included remote contact with the patients between their scheduled visits to the heart failure centers. Some studies reported that disease management programs that included home visits by nurses were effective in reducing recurrent hospital admissions or deaths among patients recruited after recent hospitalization for heart failure [21–24]. However, other studies failed to show the efficacy of such interventions [6, 25–27].

Although most patients in our study had at least one hospital admission during follow-up, fewer than half were hospitalized because of heart failure, and heart failure accounted for only 30% of the total hospital admissions. Mejhert et al. reported that among Swedish patients with heart failure, non-cardiac and cardiac hospital admissions accounted for 40% and 30% of the total long-term health-care expenditure, respectively [28]. Patients with heart failure often have other comorbidities, of which the most common are hypertension, coronary artery disease and diabetes [29]. This was also the case in our study. Thus, a disease-centerd approach may not suffice to make a significant impact on all-cause hospital admissions and mortality in patients with chronic heart failure. A recent systematic review showed that case-management interventions addressing the complex needs of patients with chronic heart failure who had been previously hospitalized for heart failure exacerbation were associated with a 12-month reduction in all-cause mortality and all-cause hospital admissions [4].

In our study, disease management had no significant effect on the long-term adherence to drug therapy recommended for patients with chronic heart failure. A possible explanation may be the high proportion of patients who were already treated with these medications upon recruitment.

We found that the study intervention was highly effective in improving patients’ health-related quality of life and depression symptoms, but these improvements were not associated with objective evidence of improved physical functioning, as tested with the 6-minute walk test. It may be that the psycho-social support delivered during the frequent contacts with the disease management nurses is the main factor in these changes.

Interestingly, the diagnosis of heart failure, taken from administrative health-care data, was not confirmed in 1,452 (27.4%) patients screened for eligibility in this study. This highlights the importance of reliance on explicit diagnostic criteria, including echocardiographic examination, for the diagnosis of heart failure. A recent systematic review showed a high variability of the positive predictive value of heart failure diagnosis based on administrative data, ranging between 12% and 100% [30].

### Study limitations and strengths

This study was powered to detect a 33% reduction in the hazard for hospital admission for heart failure caused by the disease management intervention. Thus, it may be that the study sample did not provide enough statistical power to detect a smaller effect. In fact, the point estimates for the intervention effect on the total number of in-hospital days and hospital admissions due to heart failure show less than a 33% reduction, and point in this direction.

The patients assigned to usual care were evaluated by the cardiologists at the heart failure centers every 6 months during follow-up, thus some contamination of the control intervention may be possible. In addition, the follow-up assessments of the patients' NYHA classification and 6-minute walk test were performed by assessors who were not blinded to the patients' assigned intervention. Nevertheless, there was no significant difference between the two study interventions in patients’ likelihood of experiencing a clinically important improvement in the 6-minute walk test during follow-up. Thus, this measure may be less prone to biased assessments due to lack of blinding.

Our study was carried-out in an environment with universal access to high-quality health care. In other health-care systems with barriers to universal access, the study intervention might have proven more effective in reducing hospital re-admissions and deaths.

Nevertheless, this study included a large sample size with a long follow-up, had a relatively small attrition rate and complete ascertainment of the major study endpoints. The information derived from this study may apply to other countries with similar health-care systems.

## Conclusions

This comprehensive disease management program among ambulatory patients with chronic heart failure was not proven to be superior to usual care with respect to the primary study endpoint, but delayed the time until first hospital admission for heart failure and significantly improved patient-centered outcomes, i.e. health-related quality of life and depression. Further research is needed to assess the efficacy of alternative clinical service organization methods in improving the outcomes of ambulatory patients with chronic heart failure.

## Additional file

Additional file 1: Study protocol, list of protocol changes and statistical analysis plan. (PDF 1894 kb)

## Abbreviations

ACE-I: angiotensin-converting enzyme inhibitor
ARB: Angiotensin receptor blocker
BNP: Brain natriuretic peptide
CHF: Congestive heart failure
CI: Confidence interval
HR: Hazard ratio
IQR: Interquartile range
NYHA: New York Heart Association
OR: Odds ratio
PHQ-9: Nine-item patient health depression scale
RR: Rate ratio
s.d.: Standard deviation
SF-36: 36-item short form questionnaire
