# Supplementary material for: Disease management in the treatment of patients with chronic heart failure who have universal access to health care: a randomized controlled trial
Source: BMC Med. 2017 May 1;15:90. doi: 10.1186/s12916-017-0855-z (PMC5410698; doi:10.1186/s12916-017-0855-z)
Supplement: Additional file 1: — Study protocol, list of protocol changes and statistical analysis plan. (PDF 1894 kb) [file 12916_2017_855_MOESM1_ESM.pdf]

TABLE OF CONTENTS

Document name	Page numbers
1. Original protocol	2-49
2. Summary of changes	50-51
3. Original (final) statistical analysis plan	52-59

Comprehensive Disease Management Program for Community-Dwelling Patients with Heart Failure:

Study Protocol: version-1, July 4th, 2007.

Background and rationale

The prevalence of heart failure increases with age, and is associated with high mortality rate (1, 2). A national survey conducted in internal medicine and cardiology departments in Israel in 2003 showed that in-hospital mortality among patients with diagnosis of heart failure was 4.7%, 30-day mortality was 7.7%, and 6-month mortality was 18.9% (3). Among cardiovascular diseases, heart failure is the only disease with increasing prevalence in developed countries, due to population aging and increased survival from underlying diseases (e.g. acute coronary events) (4). This increase is associated with high frequency of recurrent hospital admissions (5-8). In the United States, heart failure is the most frequent diagnosis among hospitalized patients ≥65 years of age (9). In a survey conducted in internal medicine departments in a large medical centre in Israel, heart failure was the third most frequent cause of hospital admission, accounting for 5.4% of all admissions (10). Over the years, mortality rates due to heart failure have declined in many countries, due to improved treatment (4, 6, 7, 11, 12). Nevertheless, one-year mortality among patients admitted to hospital due to heart failure was 10-times as high as the mortality in the general population (13). Hospital admissions are the main contributors to the high healthcare costs in patients with heart failure (14). In order to cope with the high rate of recurrent hospital admissions among patients with heart failure, Rich et al. evaluated in a randomized clinical trial, an innovative approach of disease management program led by nurses. The program included self-care education delivered to the patient and his\her family caregivers, psychosocial support, coordinated hospital discharge plan, nutritional advice, assessment of medical treatment and close follow-up. This break-through study showed, that compared to usual care, the program significantly reduced the number of recurrent hospital admissions due to heart failure (and subsequently the disease-related costs), and was associated with improved health-related quality of life and survival (15). Since then, many other investigators have reported the results of various disease management interventions delivered to patients with heart failure. These disease

management activities were usually delivered by healthcare professionals (most frequently nurses) trained in the management of patients with heart failure, and focused on various care components, for example: self-care education and adherence to medical therapy. These programs employed various methods, including: home visits or telephone contact with the patients. In a systematic review of 33 randomized controlled trials, comprehensive disease management programs caused a 20% reduction in mortality and 42% reduction in hospital admissions due to heart failure. A sensitivity analysis showed that the various programs showed similar effectiveness, and thus it was concluded that the selection of a specific program should be based on the characteristics of the local healthcare system, patient population and available resources (16). The clinical guidelines of the European Society of Cardiology state that an organized system of specialist heart failure care has high level of evidence for causing reduction of mortality and recurrent hospital admissions among patients with heart failure (17). The American Heart Association\American College of Cardiology clinical guidelines also recommend that care for patients with heart failure who are at high risk for recurrent hospital admissions will be delivered employing multi-disciplinary programs in order to increase adherence to recommended treatment, overcome barriers for behavioural change and reduce recurrent hospital admissions (18). Over the last few years there has been an increase in utilization of telemedicine as part of comprehensive disease management programs for patients with heart failure. The technologies used included video-sessions with the patients that substitute home visits, and use of home tele-monitoring equipment for measurements of physiological parameters such as body weight, heart rate and blood pressure, that are automatically integrated in the patients' electronic medical record (19-23). In Israel, Roth et al. reported the use of telemedicine to monitor blood pressure and body weight among patients with heart failure and a SHAHAL subscription (private payed-for service for patients with heart disease). Compared to the pre-intervention period, patients enrolled in the home tele-monitoring intervention experienced a decline in the number of hospital admissions and in-hospital days,

and improvement in health-related quality of life (24). It should be noted that this study did not include a control group.

Study objectives

1. To evaluate the feasibility of a comprehensive program that includes disease management by multidisciplinary teams operating in regional heart failure centres and heart failure nurse specialists operating in a central call centre, and home tele-monitoring of body weight, blood pressure and heart rate among patients with heart failure.
2. To evaluate the effect of this program on:
    a. Reduction of recurrent hospital admissions due to heart failure;
    b. Reduction of mortality from all causes;
    c. Improvement of health-related quality of life
    d. Improvement of the patients' functional status

The program will also include economic evaluation.

Study hypotheses

- The proportion of patients who will have the primary composite outcome (hospital admission due to heart failure or death from all causes) will be smaller than this proportion among patients assigned to the control treatment during follow-up;
- Health-related quality of life and depression scores will be better than those measured among patients assigned to the control treatment during follow-up (secondary outcome measures);
- The functional status of patients assigned to the disease management will be better than among patients assigned to the control treatment during follow-up (secondary outcome measure);

Study design

Open-label, controlled clinical trial of parallel group design.

Study target population

1,200 adult patients insured by Maccabi Health Services, with heart failure; New-York Heart Association (NYHA) functional class: II-IV

Inclusion criteria

A. Inclusion criteria for patients screened within the first two months after hospital discharge for heart failure (re: index hospitalization):

1. Men and women; age: 18 years or older;
2. The patient has relevant echocardiographic evaluation* performed within 3 months prior to the first evaluation visit in the heart failure centre;
3. The patient was discharged from hospitalization for acute, newly diagnosed heart failure, or exacerbation of existing heart failure, or decompensated heart failure during a hospital admission for other cause (information will be obtained from the discharge summary of the index hospital admission);
4. The patient has heart failure according to the ESC guidelines (ref. 17);
5. The patient had NYHA function classification II-IV at first evaluation visit (ref. 17) at ;

**-If the patient had an acute myocardial infarction during the 3 months prior to the screening visit, an updated echocardiographic evaluation has to be performed even if less than 3 months elapsed from the prior assessment.*

B. Inclusion criteria for patients who have not been hospitalized for heart failure within the first two months:

1. Age 18 years or older;
2. The patient has relevant echocardiographic evaluation* performed within 3 months prior to the first evaluation visit in the heart failure centre;
3. The patient has heart failure according to the ESC guidelines (ref. 17);
4. The patient meets at least **one** of the following criteria:
    a. Daily furosemide dose greater than 40 mg;
    b. Chronic treatment with two types of diuretic drugs;
    c. NYHA functional class IV;
    d. Six-minute walking distance <300 meter;
    e. Combined left- and right-heart failure;
    f. At least two hospital admissions for heart failure within the past 12 months;

**-If the patient had an acute myocardial infarction during the 3 months prior to the screening visit, an updated echocardiographic evaluation has to be performed even if less than 3 months elapsed from the prior assessment.*

The patient has to sign an informed consent form prior to inclusion

Exclusion criteria

1. The patient has a severe co-morbidity (for example: severe or end-stage pulmonary, renal or liver failure; metastatic cancer), which may impair his\her 3-year survival prospects;
2. The patient has severe functional impairment which is unrelated to heart failure, for example: amyotrophic lateral sclerosis, paraplegia, etc.
3. The patient cannot understand or act according to the instructions delivered by the nurse at the central call centre, or use the tele-metric equipment, or answer the study questionnaires;
4. Substance abuse (i.e. alcohol or drugs);

Recruitment of patients to the study

Candidate patients will be recruited to the trial in two ways:

- **Hospital track:** Maccabi Health Services nurse monitors operating in all public hospitals in Israel will contact patients hospitalized for heart failure (either acute newly diagnosed or exacerbation of previously diagnosed heart failure), and give them both oral and written information on the heart failure study and its objectives, and refer them to the heart failure centre closest to their place of living. Primary practitioners will be able to refer patients within 2 months of hospital discharge in cases where contact has not been made by the Maccabi nurse monitor.
- **Community track:** Cardiologists and primary practitioners in the community will be able to refer patients with previous diagnosis of heart failure if they meet the above inclusion criteria for patients who have not had heart failure hospital admission within the past 2 months.

The study intervention tested: comprehensive disease management program for community-dwelling patients with heart failure.

The intervention includes 3 elements:

1. Regional heart failure centres;
2. Central call centre
3. Home tele-monitoring

Heart failure centres

For this study, Maccabi Health Services will establish regional heart failure centres. The centres will be manned by teams of cardiologists and nurses experienced in management of patients with heart failure. The heart failure centres will meet the following clinical needs:

- Assessment of patients at recruitment and classification as stable or unstable;
- Delineation of care plan at entry;
- Set patient-specific range for body weight, blood pressure and heart rate, and thresholds for patient-specific automatic alerts;
- Clinical follow-up and updating the care plan as needed;
- Patient education delivered by nurses;

Disease Management Protocols

Designated Disease Management protocols have been created for the study to guide the Disease Management nurses in the management of patients assigned to the study intervention. These protocols include the following components: patient education for self-care; monitoring patient adherence to medical treatment and drug therapy side effects; identifying and management of acute events; indications for modification of drug therapy; instructions for patient referral; and detailed description of the circumstance where the Disease Management nurse should contact the cardiologist or the primary practitioner to get instructions for further action.

Central call centre

For this study, 'Maccabi Health Services' will set a central designated call centre that will be operated by a team of nurses experienced in management of patients with heart failure, 8 hours\day (08:00-16:00). This call centre will be used for the management of patients between their visits to the heart failure centres or their primary practitioners. Disease Management will be carried out according to the patient's treatment plan recommended by the cardiologist, the designated Disease Management protocols, and the information received from the patient's tele-monitoring. When needed, an on-call consultant cardiologist will telephone advice to the Disease Management nurses. Disease Management activities will be coordinated vis-à-vis the primary practitioner.

Tele-Monitoring

Patients assigned to the study intervention will be provided with home tele-monitoring devices for monitoring body weight, blood pressure and heart rate. These devices will transmit the tele-monitoring signal via Wi-Fi server and a telephone line at the patient's home to a server in the central call centre, where it will be automatically entered in the patient's electronic medical record. If the tele-monitoring results will be outside the patient-specific pre-set range, an automatic alert will appear. These alerts will be addressed by the central call centre nurses, according to the patient care plan assigned by the cardiologist and the Disease Management protocols.

Disease Management components delivered by the nurses between patient follow-up visits

Between follow-up visits to the heart failure centres, the nurses will manage the patients' heart failure from the call centre, via telephone calls. The telephone contact with the patients will include:

- Planned calls initiated by the nurse;

- Unplanned calls initiated by the nurse in case of an acute change in the patient's status (e.g. following hospital discharge, abnormal tele-monitoring results);
- Patient-initiated calls to the nurse;

Objectives of planned calls initiated by the nurse:

- Monitoring patient adherence to drug treatment and adverse effects of drug therapy;
- Monitoring patient adherence to lifestyle advice (diet and physical activity).
- Monitoring smoking cessation progress for patients who are smokers, and provision of smoking cessation advice and support;
- Patient education for self-care and self-monitoring;
- Referral for periodic blood tests;
- Titration of medical therapy according to the treatment plan set by the cardiologist at the heart failure centre;
- Discussing problems raised by the patient, including provision of advice for coping with the disease;
- Updating information on recent hospital admissions, emergency room visits, visits to the primary practitioner and cardiologist;

Objectives of unplanned calls initiated by the nurse:

- Verification and adaptation of drug therapy following tele-monitoring signal alert;
- Verification and adaptation of treatment after an abnormal blood test result (according to written protocols and in coordination with the primary practitioner);

Patient-initiated calls to the nurse:

- During working hours of the call-centre, the study nurse will provide advice in response to disease-related complaints or problems, i.e. exacerbation of disease-related symptoms, difficulties in operating the tele-monitoring equipment, etc.;

- During the call centre off-hours, the patients will be instructed to call the central nurse (general) call centre of 'Maccabi Health Services' ('Non-Stop Maccabi'). The nurses at this call centre will contact an on-call Disease Management nurse where needed;

Definitions: Unstable/ stable patient

- A patient is defined 'stable' if during a visit to the heart failure centre, a cardiologist decides that the patient's next visit to the heart failure centre should be scheduled in less than one month or later; Disease Management between the visits to the cardiologists at the heart failure or the primary practitioner will be conducted by the nurse at the central call centre;
- A patient is defined 'unstable' if during a visit to the heart failure centre, a cardiologist decides that the patient's next visit to the heart failure centre should be scheduled in less than one month (for example: the patient should be seen every week). In such cases, the Disease Management between the visits to the cardiologists at the heart failure or the primary practitioner will be conducted by the nurse at the heart failure centre;
- A patient who is defined 'unstable' at admission can be later defined 'stable' and vice versa. In such cases, the responsibility of disease management between visits will be transferred from the nurse at the heart failure centre to the nurse at the central call centre, or from the nurse at the central call centre to the nurse at the heart failure centre, respectively. The nurse at the heart failure centre will be responsible to make and document this transfer (see Appendix B).
- A patient who will be readmitted to the hospital during the study will be re-evaluated after discharge at the heart failure centre, and his/her status (stable/unstable) will be updated by the cardiologist (see Appendix B).

Care delivered to patients assigned to the control group

After enrolment in the study, a patient assigned to the control group will receive self-care instructions in small groups, and a digital weight scale for daily monitoring his/her body weight. The patient will also receive a treatment plan to be transferred to his/her

primary practitioner. Further care will be provided by the primary practitioner and consultant cardiologist in the community. The patient will be evaluated every 6 months by a cardiologist at the heart failure centre and his/her treatment plan will be updated. The frequency of follow-up visits to the primary practitioner or the consultant cardiologist in the community will be determined by these doctors according to the patient's needs.

Follow-up visits and study assessments

In addition to provision of care for patients assigned to the study intervention, the heart failure centres will collect baseline and follow-up information for the study. The information will be collected uniformly for all the study participants, and will include:

- Eligibility assessment of potential candidates for enrolment in the study, recruitment and random allocation to one of the two study arms;
- Data collection at baseline for study participants;
- Data collection on process and outcome variables every 6 months during follow-up until the end of the study;

Information collected for assessment upon recruitment (see also Appendix C)

Information will be collected by the cardiologists and nurses at the heart failure centres, including:

- Patients' socio-demographic characteristics (sex, age, education, family status, etc.);
- Lifestyle characteristics (cigarette smoking habits, alcohol consumption, physical activity);
- The etiologic basis for the patient's heart failure (e.g. ischemic heart disease, cardiomyopathy, valvular disease, etc.);
- Risk factors for heart failure (e.g. diabetes, hypertension, hyperlipidaemia, obesity, etc.);
- Last hospital admission for heart failure;
- Confirmation of heart failure diagnosis according to the definition of European Society of Cardiology and the American Heart Association;

- Information on left ventricular ejection fraction according to *up-to-date and relevant** echocardiographic assessment;
- Relevant biochemical and haematological parameters (e.g. renal function tests, complete blood count, blood lipid profile, etc. as depicted in Appendix C);
- Confirmation of eligibility criteria;
- Confirmation of the absence of any exclusion criterion;
- Comorbidity;
- Chronic medical therapy;
- Assessment of the patient's functional status, tested with 6-minute walk-test;
- Patient-filled health-related quality of life questionnaire (SF-36; see Appendix D);
- Assessment of depression symptoms with the PHQ-9 questionnaire;

**For eligibility assessment, an echocardiographic evaluation performed within the last three months is required. In the event of recent hospitalization for acute myocardial infarction, an echocardiographic evaluation performed after discharge is required, even if a pre-hospitalization echocardiographic assessment performed within the last 3 months is available.*

Information collected during follow-up

This evaluation will be carried out every six months, and will include:

- Updating the New York Heart Association (NYHA) function classification;
- Updating information on chronic medical therapy;
- Biochemical and haematological relevant measurements (renal function tests, complete blood count, blood lipid profile, etc. see Appendix E);
- Assessment of the patient's functional status, tested with 6-minute walk-test;
- Patient-filled health-related quality of life questionnaire (SF-36; see Appendix D);
- Assessment of depression symptoms with the PHQ-9 questionnaire;
- Information on hospital admissions within the last six months;

- Information on visits to emergency room and acute care centres due to exacerbations of heart failure;
- For patients assigned to the study intervention: assessment of utilization of home tele-monitoring equipment;

Study Outcome Variables

- ***Main outcome composite variable***: First hospital admission for heart failure due to acute exacerbation of heart failure or death from any cause;
- ***Secondary outcome variables***:
  - Health-related quality of life (SF-36) and depression (PHQ-9);
  - Functional status (assessed with NYHA classification and 6-min. walk-test);
  - Utilization of health services:
    - Hospital admissions;
    - Visits to emergency room and acute care services;
    - Visits to cardiologists;
    - Visits to primary practitioners;
    - Visits to nurses at the primary care clinics and at the heart failure centres;
    - Medical treatment (dosage) of drugs for heart failure (recommended in clinical guidelines);
    - Referrals to social workers and dietitians;
    - Utilization of imaging tests;
    - Utilization of laboratory tests;

Description of study tools, their reliability and validity

Assessment of quality of life parameters will include:

1. A self-administered generic questionnaire the 36-Short Form Health Survey (SF-36) (25). This questionnaire was validated for the Israeli population (26), and is extensively used for assessment of various health conditions, including heart failure (27). In addition, this questionnaire was found to have

a better discriminative capacity between the emotional and physical components of health-related quality of life compared to a heart-failure specific quality of life questionnaire (Minnesota Living with Heart Failure Questionnaire) (27).

2. Utility assessment, using Visual Analog Scale (VAS). The patient will be asked to rate his health status on a scale ranging between 0 and 100, where 0 is given for death and 100 for excellent health (27, 28). This assessment will support cost-utility analysis.

Functional status will be assessed with two measures:

1. New York Heart Association (NYHA) functional classification (17). According to this classification, heart failure severity is classified in 4 categories, where patients who are asymptomatic while performing usual physical efforts are at NYHA class-I, and patients who have symptoms of heart failure at-rest that exacerbate during minimal effort are classified as having NYHA-IV heart failure (see Appendix B). This classification is widely used in studies in cardiology, especially in heart failure studies.
2. 6-minute walk-test: this is a relatively easily performed test, evaluating the distance that a patient is able to walk within 6 minutes, in standard conditions (29, 30). This test is commonly used in studies evaluating functional status of patients with heart failure, and is an independent prognostic measure for these patients (30).

Study sample size and sample size and structure justification

Outcome: All-cause mortality at 12 months

From the paper by Roccaforte et al (16), we assume that the odds ratio is 0.80. We assume that 18% of usual care patients will die within the first 12 months.

The statistical power for detecting a significant difference at the 5% level (two-tailed test) is:

- For 600 patients in the usual care and 600 in the experimental care group: 32%
- For 400 patients in the usual care and 800 in the experimental care group: 30%

Conclusion:

There is not sufficient statistical power to include this outcome as one of the major endpoints of interest.

Approximately 4500 patients would be required to study this outcome with sufficient statistical power.

Randomization in a 2:1 ratio instead of 1:1 ratio is equivalent to reducing the sample size by about 13%.

Outcome: Re-hospitalization for heart disease at 12 months

From the paper by Roccaforte et al (16), we assume that the odds ratio is either 0.58 (their point estimate) or 0.67 (their upper 95% confidence limit). We assume that 33% of usual care patients will be re-hospitalized for heart disease within the first 12 months.

The statistical power for detecting a significant difference at the 5% level (two-tailed test), when the odds ratio is 0.58 is:

- For 600 patients in the usual care and 600 in the experimental care group: 99%
- For 400 patients in the usual care and 800 in the experimental care group: 98%

The statistical power for detecting a significant difference at the 5% level (two-tailed test), when the odds ratio is 0.67 is:

- For 600 patients in the usual care and 600 in the experimental care group: 89%
- For 400 patients in the usual care and 800 in the experimental care group: 85%

Conclusion:

There is sufficient statistical power to include this outcome as one of the major endpoints of interest.

Randomization in a 2:1 ratio instead of 1:1 ratio loses some power, but still provides acceptable power for this sample size.

Because re-hospitalization for heart disease is affected by early mortality, it is worth considering a joint mortality/re-hospitalization outcome. One measure to consider would be the percentage of patients either re-hospitalized for heart disease or dying from any cause within the first 12 months. Although we do not currently have data to do sample size calculations for this outcome, the results are expected to be similar to those given above for re-hospitalization.

Interim analysis for safety

The trial will be monitored for safety to prevent it being extended in the face of adverse results. The two main outcomes for safety will be all-cause mortality and re-hospitalization for any reason. Unlike efficacy analyses these outcomes will be treated on a continuous time scale and not simply after the 12 month period.

The Kaplan-Meier plots of time to death and time to re-hospitalization will be examined by the Data Monitoring Committee at regular periods, and log-rank tests for differences in hazard ratios will be conducted.

The Data Monitoring Committee will consider recommending premature closure of the study in the event of finding a statistically significantly higher death or re-hospitalization rate in the experimental group, where significance will be adjusted for multiple testing using a one-sided Pocock boundary.

These data will be examined every 6 months, and end at 6 months prior to the planned trial termination. If patient recruitment rates are those envisaged (100 per month for the first 4 months, and 200 per month for the following 4 months) then, allowing a 2.5 month gap for updating the data base with current information, the proportion of total follow-up time accrued at these analyses will be 6%, 20%, 44%, and 69% respectively.

The data presented and discussed in the Data Monitoring meetings will not be disclosed to the investigators, unless a recommendation for premature termination of the study is made.

Statistical analysis

a.) Main analysis

Comparison between the two interventions (i.e. Disease Management, the study intervention, and Usual Care, the control intervention) will be made by log-rank test, where an event is defined as hospital admission due to heart failure or death from any cause. In this analysis, the occurrence of any of these two events is defined as 'failure', and the time until the first event (hospital admission or death) will be defined as the time-to-failure. The analysis will be according to intention-to-treat, meaning that patients' data will be analysed according to their assigned intervention. The log-rank test will be one-sided at a significance level of 2.5%, where the alternative hypothesis is that this time will be longer in the study intervention group. Since the Data Monitoring Committee acts as a safety committee and the tests used for this purpose will be in the other direction, looking for poorer results in the study intervention group, no correction for multiple comparisons are needed.

Secondary analyses

There will be several secondary analyses to support the primary hypothesis. They will include:

(i) Comparisons of baseline factors among the two study groups, e.g. age, NYHA functional classification, 6-min. walk test, left ventricular ejection fraction, type of heart failure, plasma creatinine and heart failure center;
(ii) Adjustment for these baseline factors will be made using the Cox proportional hazards model;
(iii) An interaction between the NYHA functional classification and study group will be tested;
(iv) An interaction between the time of recruitment and study group will be tested, since we expect a 'learning effect' in operating the study intervention;

In addition, the two study groups will be compared for the other secondary outcomes, for example:

(i) The number of hospital admissions for heart failure per year;
(ii) Death from all causes;
(iii) NYHA classification tested every 6 months during follow-up;
(iv) Health related quality of life and depression symptoms tested every 6 months during follow-up;

Total costs will be compared between the two interventions.

The statistical methods for these comparisons will be chosen according to the distribution of each outcome (categorical, ordered or continuous). Each outcome will be tested in univariate and multiple variable models.

Potential bias and ways to reduce its effect

The fact that the efficacy of the study intervention will be evaluated within a randomized controlled trial, where patients' assigned intervention will be determined by central computerized randomization process and allocation concealment will be maintained, will significantly reduce the probability for selection bias. The fact that the randomization will be stratified by heart failure centre will control for possible confounding effect of the patients' and healthcare professionals' characteristics, which may vary across heart failure centres. In any case, these characteristics will be entered into the multivariable models assessing the differences between the two study arms.

The randomization of individual patients rather than by heart failure centres (cluster randomization) increases the potential for 'contamination' of the control group by the intervention, thus the study may provide an underestimation of the 'true' effect of the study intervention. On the other hand, randomization by study centres is not considered optimal, given the small number of heart failure centres and the high level of variance among them, which cannot be well controlled for in the multivariable analyses.

Pilot Study

The first 3 months will be conducted as a pilot study where the process of patient recruitment will be tested; there will be a stepwise operation of the heart failure centres, assessment of operational problems of the patients' electronic records and the call centre, problem solving process and study tools adaptation.

Reference

1) Massie B, Shah N. Evolving trends in the epidemiologic factors of heart failure: Rationale for preventive strategies and comprehensive disease management. Am Heart J, 1997; 133:703-712.
2) Bleumink GS, Knetsch AM, Sturkenboom MCJM, et al. Quantifying the heart failure epidemic: prevalence, incidence rate, lifetime risk and prognosis of heart failure. The Rotterdam Study. Eur Heart J, 2004; 25:1614–1619.
3) HFSIS 2003. Heart Failure Survey in Israel 2003. National Survey of Hospitalized Patients March-April 2003. Preliminary Findings. Israel Center of Disease Control, 2004. Publication 234.
4) Roger VL, Weston SA, Redfield MM, et al. Trends in heart failure incidence and survival in a community-based population. JAMA, 2004; 292:344-350.
5) Ghali JK, Cooper R, Ford E. Trends in hospitalization rates for heart failure in the United States, 1973-1986: evidence for increasing population prevalence. Arch Intern Med 1990; 150:769-773.
6) Stewart S, MacIntyre K, MacLeod MMC et al. Trends in hospitalization for heart failure in Scotland, 1990–1996. Eur Heart J, 2001;22:209–17.
7) Blackledge H M, Tomlinson J, Squire I B. Prognosis for patients newly admitted to hospital with heart failure: survival trends in 12 220 index admissions in Leicestershire 1993–2001. Heart, 2003;89:615–620.
8) T P Ng, M Niti. Trends and ethnic differences in hospital admissions and mortality for congestive heart failure in the elderly in Singapore, 1991 to 1998. Heart 2003;89:865–870
9) National Center for Health Statistic, Graves EJ. 1989 Summary: national hospital discharge survey. Advanced data from vital and health statistics. No. 199. Hyattsville, Md.:Public Health Service, 199:1-12. (DHHS publication no. (PHS) 91-1250.)
10) Stein GY, Zeidman A. Trends in admissions to internal medicine wards in a community hospital in Israel. Eur J Intern Med, 2006; 17:281-285.

11) Baker DW, Einstadter D, Thomas C, Cebul R D. Mortality trends for 23,505 Medicare patients hospitalized with heart failure in Northeast Ohio, 1991 to 1997. Am Heart J 2003;146:258–64.
12) Schaufelbergera M, Swedberga K, Ko¨sterb M, Rose´nb M, Rosengrena A. Decreasing one-year mortality and hospitalization rates for heart failure in Sweden.
Data from the Swedish Hospital Discharge Registry 1988 to 2000. Eur Heart J, 2004; 25:300–307.

13) Shahar E, Lee S, Kim J, PhD, Duval S, Barber C, Luepker RV. Hospitalized Heart Failure: Rates and Long-Term Mortality. J Card Fail, 2004; 10: 374-379.
14) Parmley W. Cost-effective management of heart failure. Clin Cardiol, 1996;19:240–242.
15) Rich MW, Beckham V, Wittenberg C, Leven CL, Freedland KE, Carney RM. A multidisciplinary intervention to prevent the readmission of elderly patients with congestive heart failure. N Engl J Med,1995; 333:1190-1195.
16) Roccaforte R, Demers C, Baldassarre F, Teo KK, Yusuf S. Effectiveness of comprehensive disease management programmes in improving clinical outcomes in heart failure patients. A meta-analysis. Eur J Heart Fail, 2005; 7:1133-1144.
17) The Task Force for the diagnosis and treatment of CHF of the European Society of Cardiology. Guidelines for the diagnosis and treatment of Chronic Heart Failure: full text (update 2005). Eur Heart J, 2005; doi:10.1093/eurheartj/ehi205:1-45.
18) ACC/AHA Task Force on Practice Guidelines. ACC/AHA 2005 Guideline Update for the Diagnosis and Management of Chronic Heart Failure in the Adult- Summary Article. Circulation, 2005; 112:1825-1852.
19) Benatar D, Bondmass M, Ghitelman J, Avital B. Outcomes of chronic heart failure. Arch Intern Med, 2003; 163:347-352.
20) Mortara A, Pinna GD, Johnson P, Dargie H, La Rovere MT, Ponikovski P, Tavazzi L, Sleight P, on behalf of the HHH Investigators. A multi-country

randomized trial of the role of a new telemonitoring system in CHF: the HHH study (Home or Hospital in Heart Failure). Rationale, study design and protocol. Eur Heart J, 2004; 6 (suppl F), F99-F102.

21) Mortara A. A multi-country randomized trial on the role of a new telemonitoring system in CHF: the HHH Study (Home or Hospital Heart Failure). Presented at the European Society of Cardiology congress in Barcelona, September, 2006. http://www.escardio.org/knowledge/OnlineLearning/webcast/congress2006/Hot_Line_I.htm

22) Galbreath AD, Krasuski RA, Smith B, Stajduhar KC, Kwan MD, Ellis R, Freeman GL. Long-term heathcare and cost outcomes of disease management in a large, randomized, community-based population with heart failure. Circulation, 2004; 110:3518-3526.

23) Cleland JGF, Louis AA, Rigby AS, Janssens U, Balk AHMM, on behalf of the TEN-HMS Investigators. Noninvasive home telemonitoring for patients with heart failure at high risk of recurrent admission and death. The Trans-European Network-Home-Care Management System (TEN-HMS) Study. J Am Coll Cardiol, 2005; 45:1654-1664.

24) Roth A, Kajiloti I, Elkayam I, Sander J, Kehati M, Golovner M. Telecardiology for patients with heart failure: the 'SHL' experience in Israel. Int J Cardiol, 2004; 97:49-55.

25) Ware JE, Snow KK, Kosinski M, Gandek B. SF-36 Health Survey. Manual and interpretation Guide. The Health Institute, New England Medical Center. Boston, MA, 1993.

26) Lewin-Epstein N, Sagiv-Schifter T, Shabtal EL, Shmueli A. Validation of the 36-Item Short-Form health survey (Hebrew version) in the adult population of Israel. Med Care, 1998; 36:1361-1370.

27) Sneed NV, Paul S, Michel Y, VanBakel A, Hendrix G. Evaluation of 3 quality of life measurement tools in patients with chronic heart failure. Heart Lung, 2001; 30:332-340.

28) Shafazand S, Goldstein MK, Doyle RL, Hlatky MA, Gould MK. Health-related quality of life in patients with pulmonary arterial hypertension. Chest, 2004; 126:1452-1459.
29) Fleg JL, Piña IL, Balady GJ, et al. Assessment of functional capacity in clinical and research applications. An advisory from the Committees on Exercise, Rehabilitation and Prevention, Council on Clinical Cardiology, American Heart Association. Circulation 2000; 102:1591–7.
30) Willenheimer R, Erhardt LR. Value of 6-min-walk test for assessment of severity and prognosis of heart failure. Lancet 2000; 355:515– 6.
31) Andrea Passantino, Rocco Lagioia, Filippo Mastropasqua, Domenico Scrutinio. Short-term change in distance walked in 6 min. is an indicator of outcome in patients with chronic heart failure in clinical practice. J Am Coll Cardiol 2006; 48:99 –105.

Appendix-A: Patient recruitment and follow-up chart

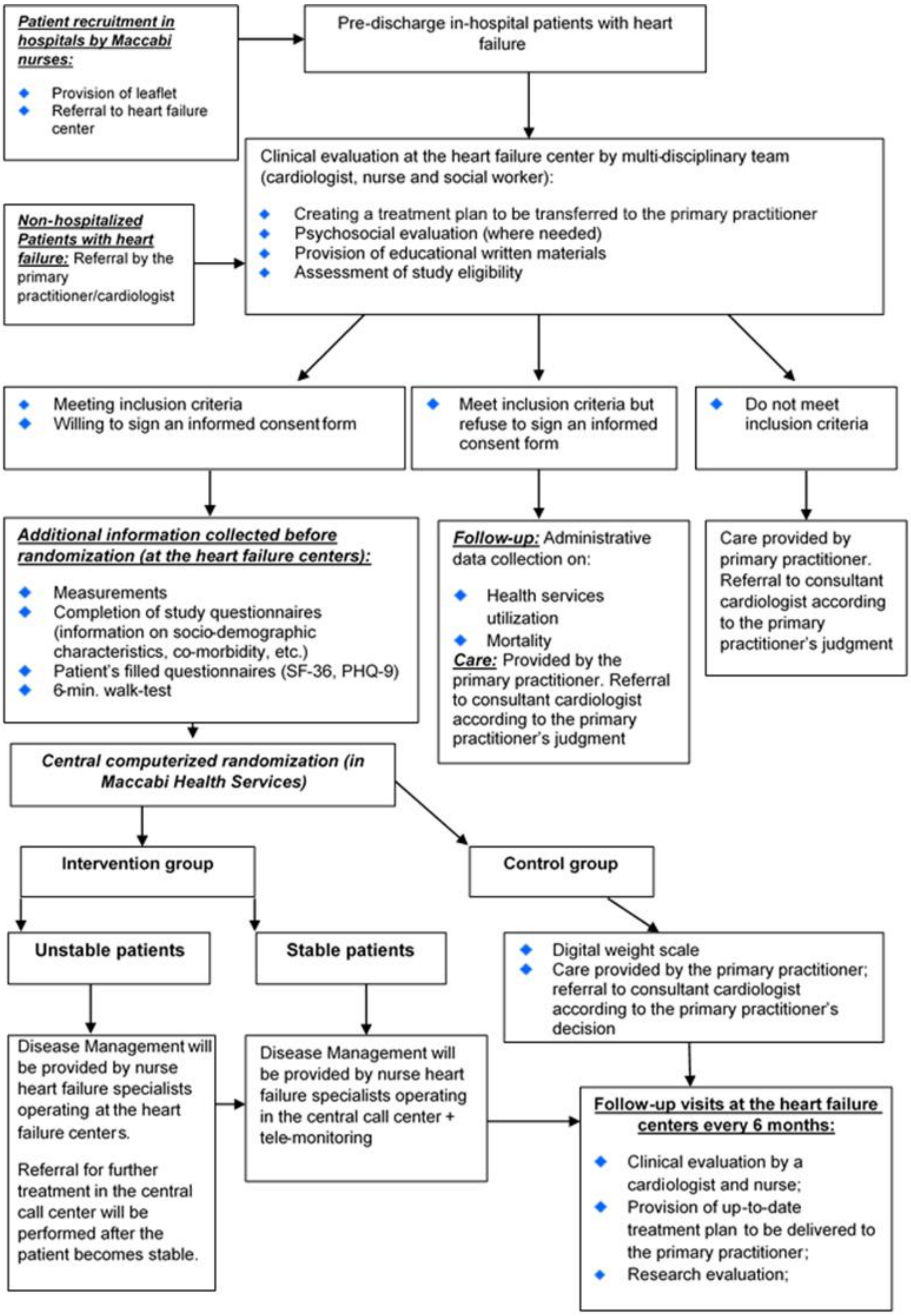

Appendix B: Delineation of patient-treatment in the study intervention arm

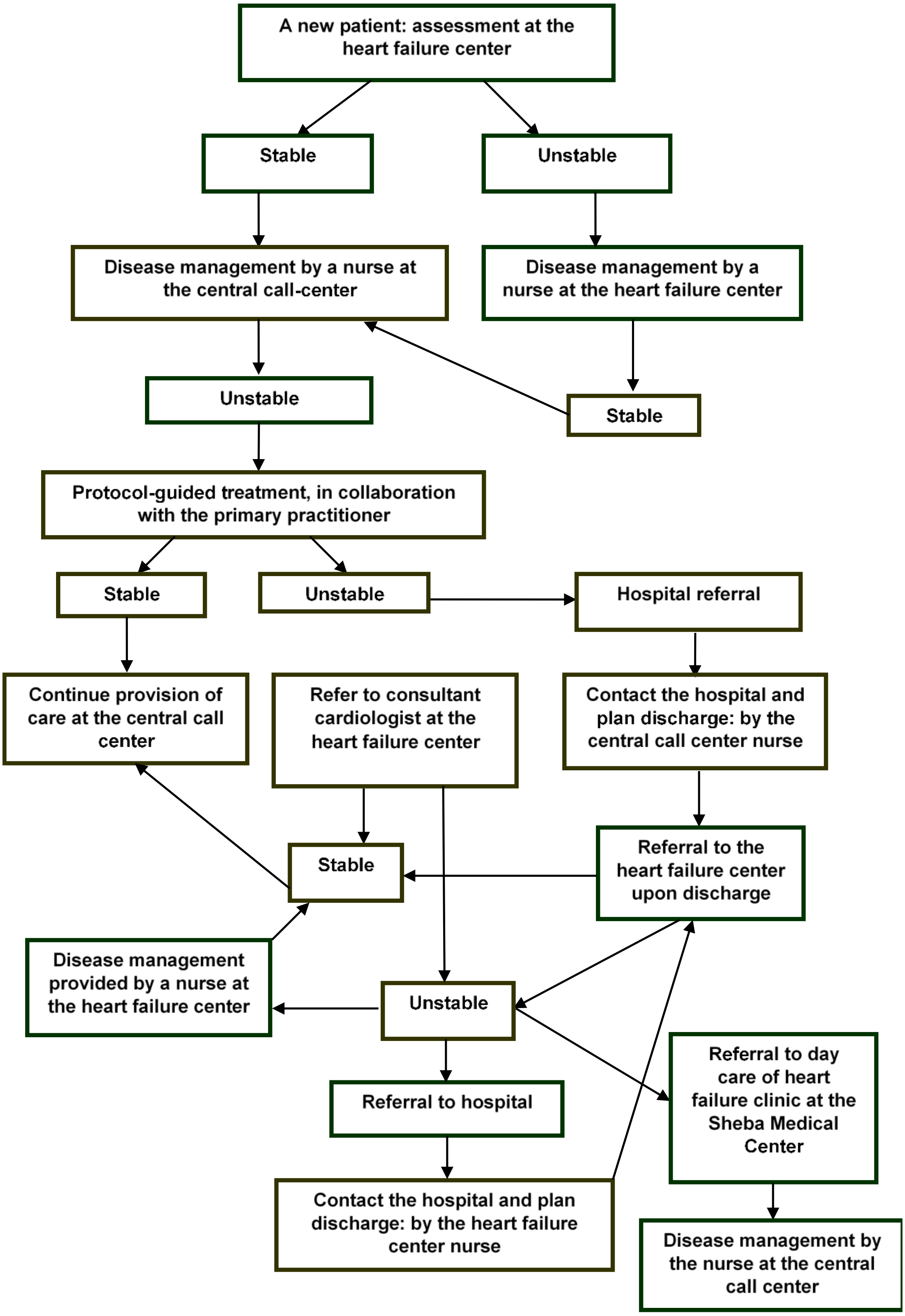

Appendix-C: Baseline assessment

Heart failure centre ______________________________|_|

I. Demographic information

Patient name: _________ __________ I.D. Number |_|_|_|_|_|_|_|_|_| -|_|

Sex: male/female Birth date: |_|_| - |_|_| - |_|_|_|_|

Land of birth: ___________________|_|_|_| **Year of immigration:** |_|_|_|_|

Address: ____ Zip: |_|_|_|_|_|

Home telephone number: |_|_| - |_|_|_|_|_|_|_|

Mobile phone number: |_|_|_| -|_|_|_|_|_|_|_|

1. Family status:

   a. Married

   b. Widower

   c. Divorced

   d. Bachelor

2. Living condition:

   a. Lives at home

   b. Lives in old-age home

   c. Other, please specify:____________________ |_|_|

3. Who are you living with: (allow multiple answers)

   a. With spouse

   b. With son/daughter

   c. With other (non-family member) care-giver

   d. With other person, please specify: _________________|_|_|

   e. Lives alone

4. Number of years of formal education |__|__| years

5. Are you working?

    a. Yes (move to question 7)

    b. No

6. Why don't you work?

    a. Retired

    b. Unemployed

    c. Due to ill health, please specify: ________________|__|__|__|

    d. Other, please specify: ________________|__|__|__|

7. Do you have a profession?

    a. Yes

    b. No (move to section II)

8. What is your profession? ______________________|__|__|__|

II. Referral to the heart failure centre

1. First visit date to the heart failure centre: |__|__| - |__|__|- |__|__|__|__|
2. Type of referral:
    a. Referred after hospital admission by Maccabi nurse at the hospital
    b. Referred by a primary practitioner, within two months after a hospital discharge
    c. Referred by a consultant cardiologist, within two months after a hospital discharge
    d. Was hospitalized more than 2 months ago, referred by the primary practitioner (move to section III, question-1)
    e. Was hospitalized more than 2 months ago, referred by the consultant cardiologist (move to section III, question-1)
    f. Other, please specify________________________________|__|

3. Information on the hospitalization that lead to the patient referral (a copy of the discharge summary should be transferred to the Gertner Institute)

3a. Hospital admission date: |__|__| - |__|__|- |__|__|__|__|

3b. Hospital discharge date: |__|__| - |__|__|- |__|__|__|__|

3c. Hospital ____________|__|__|__| Discharge ward ________________|__|__|

3d. Discharge diagnoses:

Diagnosis	***ICD-9 code***
**1.**	|__|__|__| . |__|__|
**2.**	|__|__|__| . |__|__|
**3.**	|__|__|__| . |__|__|
**4.**	|__|__|__| . |__|__|
**5.**	|__|__|__| . |__|__|
**6.**	|__|__|__| . |__|__|
**7.**	|__|__|__| . |__|__|
**8.**	|__|__|__| . |__|__|
**9.**	|__|__|__| . |__|__|
**10.**	|__|__|__| . |__|__|
**11.**	|__|__|__| . |__|__|
**12.**	|__|__|__| . |__|__|

III. Clinical evaluation at baseline: patient history

1. Heart failure aetiology

Secondary	Primary	*Mark √ where appropriate. Please note where there is a complex etiology. In this case please indicate which is the primary and which is the secondary aetiology*	
|__|	|__|	**1. Coronary Artery Disease**	|__|
|__|	|__|	**2. Hypertensive Heart Disease**	|__|
|__|	|__|	**3. Pericardial Disease**	|__|
|__|	|__|	**4. Cor pulmonale**	|__|
|__|	|__|	**5. Diabetic**	|__|
|__|	|__|	**6. Congenital Heart Disease**	|__|
|__|	|__|	7. **Cardiomyopathy:**	|__|
|__|	|__|	7a. Dilated	|__|
|__|	|__|	7b. Familial	|__|
|__|	|__|	7c. Hypertrophic: Evidence for obstruction? 1. Yes; 2. No	|__|
|__|	|__|	7d. Post-myocarditis	|__|
|__|	|__|	7e. Alcoholic	|__|
|__|	|__|	7f. Restrictive	|__|
|__|	|__|	7g. Infiltrative, specify: ____________ |__|	|__|
|__|	|__|	7h. Cytotoxic (Adriamycin)	|__|
|__|	|__|	7i. Other, specify: ____________ |__|	|__|
|__|	|__|	8. **Valvular Disease**:	|__|
|__|	|__|	8a. Rheumatic	|__|
|__|	|__|	8b. Degenerative	|__|
|__|	|__|	8c. Ischemic	|__|
|__|	|__|	8d. Atherosclerotic	|__|
|__|	|__|	8e. Bacterial endocarditis	|__|

2. Risk Factors
   a. Smoking status:
      I. Is the patient a current or past smoker?
         1. Yes, current
         2. Yes, but hasn't smoked during the last 6 months or longer;
         3. No (move to question 3b.)
      II. If positive, how many cigarettes per day? |__|__|__|; if less than 1 cigarette/day, write '00'
      III. Age at smoking initiation: |__|__|__| years;
      IV. Overall, how many years has he/she been smoking? |__|__| years

   b. Alcohol consumption

One drink = one can of beer (330cc); or one glass of wine (120 cc); or one shot of spirits (e.g. Vodka, whiskey, cognac) (40 cc)

Number of alcohol units per day: |__|__|.|__|__|; if drinks less than one unit a day write mean number of units per day according to the number of units per week divided by seven. If drinks less than one unit per week, write '00';

   c. ***Hypertension***:
      1. Yes 2. No
   d. ***Dyslipidaemia***:
      1. Yes 2. No
   e. Diabetes
      1. Yes 2. No
   f. ***Menopause*** (for women):
      1. Yes 2. No
   g. ***Chronic obstructive lung disease*** (COPD)
      1. Yes 2. No
   h. S/P ***acute myocardial infarction***
      1. Yes 2. No

i. S/P ***PCI***

1. Yes 2. No

j. S/P ***CABG***

1. Yes 2. No

k. ***Peripheral vascular disease*** (PVD)

1. Yes 2. No

l. Stroke/TIA

1. Yes 2. No

m. History among first degree relatives:

a. Coronary artery disease (<60 years of age for men, or <50 for women);

b. Cardiomyopathy;

c. Sudden death (<60 years of age for men, or <50 for women);

3. Current chronic medical therapy

<table>
<tr><th>Drug</th><th>Dose</th><th>ATC code</th></tr>
<tr><td>1.</td><td>|__|__|__| mg/gr/units X |__| /day</td><td>|__|__|__|__|__|</td></tr>
<tr><td>2.</td><td>|__|__|__| mg/gr/units X |__| /day</td><td>|__|__|__|__|__|</td></tr>
<tr><td>3.</td><td>|__|__|__| mg/gr/units X |__| /day</td><td>|__|__|__|__|__|</td></tr>
<tr><td>4.</td><td>|__|__|__| mg/gr/units X |__| /day</td><td>|__|__|__|__|__|</td></tr>
<tr><td>5.</td><td>|__|__|__| mg/gr/units X |__| /day</td><td>|__|__|__|__|__|</td></tr>
<tr><td>6.</td><td>|__|__|__| mg/gr/units X |__| /day</td><td>|__|__|__|__|__|</td></tr>
<tr><td>7.</td><td>|__|__|__| mg/gr/units X |__| /day</td><td>|__|__|__|__|__|</td></tr>
<tr><td>8.</td><td>|__|__|__| mg/gr/units X |__| /day</td><td>|__|__|__|__|__|</td></tr>
<tr><td>9.</td><td>|__|__|__| mg/gr/units X |__| /day</td><td>|__|__|__|__|__|</td></tr>
<tr><td>10.</td><td>|__|__|__| mg/gr/units X |__| /day</td><td>|__|__|__|__|__|</td></tr>
</table>

IV. Baseline measurements:

1. Weight |__|__|__|.|__| kg

2. Height |__|__|__|.|__| cm

3. Waist circumference |__|__|__|.|__| cm

4. Blood pressure* |__|__|__|/|__|__|__| mmHg

**-Should be measured while the patient is sitting, after at least 5 minutes' rest, while the patient's arm rests on a horizontal surface at the level of the heart. The cuff should match the patient's arm circumference.*

5. Pulse rate* |__|__|__| min

*-Pulse should be measured at rest, after blood pressure measurement

6. Pulse regularity:

Regular/ Irregular

V. Blood tests (to be obtained every six months unless otherwise specified)

test	***Date (mm/yyyy)***	***results***
Haemoglobin	|_|_|/|_|_|_|_|	|_|_|.|_| gr/dL
Creatinine	|_|_|/|_|_|_|_|	|_|.|_| mg/dL
Urea	|_|_|/|_|_|_|_|	|_|_| mg/dL
Uric acid	|_|_|/|_|_|_|_|	|_|_| mg/dL
Na^+	|_|_|/|_|_|_|_|	|_|_|_| meq/L
K^+	|_|_|/|_|_|_|_|	|_|_|_| meq/L
Albumin	|_|_|/|_|_|_|_|	|_|.|_| mg/dL
Glucose	|_|_|/|_|_|_|_|	|_|_|_| mg/dL
*HbA1c**	|_|_|/|_|_|_|_|	|_|_|.|_|%
*Total-cholesterol***	|_|_|/|_|_|_|_|	|_|_|_| mg/dL
*LDL-cholesterol***	|_|_|/|_|_|_|_|	|_|_|_| mg/dL
*HDL-cholesterol***	|_|_|/|_|_|_|_|	|_|_|_| mg/dL
*Triglycerides***	|_|_|/|_|_|_|_|	|_|_|_| mg/dL
*FT4***	|_|_|/|_|_|_|_|	|_|.|_| mcg/dL
*TSH***	|_|_|/|_|_|_|_|	|_| mU/mL.|_|_|
*Vit B12***	|_|_|/|_|_|_|_|	|_|_|_|_| pg/mL
*Folic Acid***	|_|_|/|_|_|_|_|	|_| ng/mL.|_|_|
*Iron***	|_|_|/|_|_|_|_|	|_|_|_| mcg/dL

-For diabetic patients only

****-Once a year***

Resting Echocardiographic evaluation:

Date of test: |_|_| - |_|_| - |_|_|_|_|

Echocardiographic evaluation

1. Left atrium diameter	|_|_| mm
2. End-diastolic left ventricular diameter (LVEDD)	|_|_| mm
3. Intra-ventricular septal thickness	|_|_| mm
4. Left ventricular ejection fraction (LVEF)	|_|_| %
5. Pulmonary systolic pressure	|_|_| mmHg
6. Moderate or severe mitral insufficiency	1. Yes; 2. No;
7. Moderate or severe tricuspid insufficiency	1. Yes; 2. No;
8. Pressure gradient across the aortic valve	|_|_| mmHg<br>Valve surface area*:|_|_| cm^2<br>***-The pressure gradient should be calculated if greater than 25 mmHg***

Six minute walk test: |_|_|_| meter. (Write ‘000’ if not tested; specify the reason for the missing measurement: _______________________|_|_|)

Resting ECG:

Date of test: |_|_| - |_|_| - |_|_|_|_|

Findings:

1. QRS width |_|_| mm

**Findings** *(mark √ where appropriate)*	**No**	**Yes**
2. LBBB	|_|	|_|
3. RBBB	|_|	|_|
4. Atrial fibrillation	|_|	|_|
5. Pathological Q waves that indicate old MI	|_|	|_|

VI. Clinical evaluation summary:

1. Is there evidence for high-output heart failure? 1. Yes; 2. No

2. Does the patient have chronic atrial fibrillation? 1. Yes; 2. No

3. Characteristics of the heart failure:

    a. Mainly left heart failure; (no or minimal right heart failure);

    b. Mainly right heart failure; (no or minimal left heart failure);

    c. Combined left and right heart failure;

4. Does the patient have an implantable device?

    a. **VVI** pacemaker;

    b. Atrial-ventricular pacemaker; **DDD**

    c. Biventricular pacemaker; **CRT**

    d. Biventricular pacemaker and defibrillator (**CRTD**)

    e. Defibrillator without pacing (**ICD**)

    f. No defibrillator or pacemaker

5. Recommended medical treatment (treatment plan delivered to the primary practitioner):

Drug	***Dose***	***ATC code***
**1.**	|_|_|_| mg/gr/units X |_| /day	|_|_|_|_|_|
**2.**	|_|_|_| mg/gr/units X |_| /day	|_|_|_|_|_|
**3.**	|_|_|_| mg/gr/units X |_| /day	|_|_|_|_|_|
**4.**	|_|_|_| mg/gr/units X |_| /day	|_|_|_|_|_|
**5.**	|_|_|_| mg/gr/units X |_| /day	|_|_|_|_|_|
**6.**	|_|_|_| mg/gr/units X |_| /day	|_|_|_|_|_|
**7.**	|_|_|_| mg/gr/units X |_| /day	|_|_|_|_|_|
**8.**	|_|_|_| mg/gr/units X |_| /day	|_|_|_|_|_|
**9.**	|_|_|_| mg/gr/units X |_| /day	|_|_|_|_|_|

VII. Study eligibility check

Inclusion criteria:

I. For patients discharged from hospital up to two months before assessment for study enrolment:

1. Age 18 years or older
2. The patient was hospitalized for:
    a. newly diagnosed, acute heart failure, or
    b. exacerbation of chronic heart failure, or
    c. heart failure exacerbation complicating hospital admission for another cause;
3. Echocardiographic examination which included evaluation of left ventricular function has been performed up to 3 months prior enrolment evaluation or after hospital discharge for acute coronary syndrome, whichever comes last.
4. The patient has heart failure according to the European Society of Cardiology Guidelines (see definition below).
5. The patient has stage C or D heart failure (see definition below).
6. The patient has NYHA function class II to IV at the time of enrolment assessment (see definition below).

II. For patients recruited more than two months after hospital discharge, referred from the community

1. Age 18 years or older
2. Echocardiographic examination which included evaluation of left ventricular function has been performed up to 3 months prior enrolment evaluation or after hospital discharge for acute coronary syndrome, whichever comes last.

3. The patient has heart failure according to the European Society of Cardiology Guidelines (see definition below).
4. The patient has stage C or D heart failure (see definition below)
5. The patient has at least one of the following criteria:
    a. Chronic treatment with furosemide >40 mg/d;
    b. Chronic treatment with two types of diuretic drugs;
    c. NYHA functional class IV (see definition below).;
    d. 6-min. walk test<300 m;
    e. The patient has combined left and right ventricular heart failure;
    f. The patient had at least two hospital admissions for heart failure exacerbations within the last 12 months;

Exclusion Criteria

1. The patient has other severe morbidity which may compromise his\her survival prospects within the next 3 years, e.g. pulmonary insufficiency, end-stage liver or renal failure, metastatic malignancy.
2. The patient is bed-ridden or has severe functional impairment which is unrelated to heart failure (e.g. paraplegia, amyotrophic lateral sclerosis).
3. The patient is unable to understand or adhere to the study protocol, e.g. unable to understand the telephone instructions given by the nurse, to operate the tele-monitoring equipment, to answer the study questionnaires.
4. The patient has alcohol or drug addiction.
5. The patient is participating in another trial.
6. The patient refuses to participate in this trial or sign an informed consent form.

Definitions

Definition of Heart Failure*:

1. The patient has typical symptoms of heart failure (in rest or during exercise), and
2. The patient has an objective echocardiographic evidence of cardiac dysfunction (systolic and/or diastolic) at rest, and (in case where the diagnosis is in doubt),
3. The patient has a favourable response to treatment directed to heart failure;

Criteria 1 and 2 should be fulfilled in all patients.

* Ref. Swedberg K, Cleland J, Dargie H, Drexler H, Follath F, Komajda M, et al. Task Force for the Diagnosis and Treatment of Chronic Heart Failure of the European Society of Cardiology. Guidelines for the diagnosis and treatment of chronic heart failure: executive summary (update 2005): The Task Force for the Diagnosis and Treatment of Chronic Heart Failure of the European Society of Cardiology. *Eur Heart J*, 2005; **26:**1115-40.

Heart Failure Stage*

A. Patients at high risk of developing HF. **(Should not be included in the study)**
B. Patients who have developed structural heart disease but who have never shown signs or symptoms of HF. **(Should not be included in the study)**
C. Patients who have current or prior symptoms of HF associated with underlying structural heart disease.
D. Patients with advanced structural heart disease and marked symptoms of HF at rest despite maximal medical therapy and who require specialized interventions.

* Ref. Hunt SA, Baker DW, Chin MH, Cinquegrani MP, Feldman AM, Francis GS, et al. ACC/AHA Guidelines for the evaluation and management of chronic heart failure in the adult: Executive summary a report of the American College of Cardiology/American Heart Association Task Force on practice guidelines (Committee to revise the 1995 guidelines for the evaluation and management of heart failure). *Circulation*, 2001; **104:** 2996 –3007.

NYHA functional classification*

I. No limitations of physical activity. No heart failure symptoms;

II. Mild limitation of physical activity. Heart failure symptoms with significant exertion; comfortable at rest or with mild activity;

III. Marked limitation of physical activity. Heart failure symptoms with mild exertion; only comfortable at rest;

IV. Discomfort with any activity. Heart failure symptoms occur at rest;

*-Source: The Criteria Committee of the New York Heart Association. Diseases of the Heart and Blood Vessels: Nomenclature and Criteria for Diagnosis. 6th ed. Boston, Mass: Little Brown; 1964.

Informed consent form

A. The patient signed an informed consent form
B. The patient refuses to sign an informed consent form → ***Not eligible***

Did the patient provide filled questionnaires? (SF-36, PHQ-9)

A. Yes
B. No, please specify the reason: ______________________ |_|_|

The patient's assigned treatment (please specify the randomization result):

A. The patient is assigned to the control group (end of the questionnaire);

B. The patient is assigned to the study intervention group

The date of the next patient's follow-up visit to the cardiologist at the heart failure center*: |_|_| - |_|_| - |_|_|_|_|

*-for clinical purpose, ***only*** in the study intervention group*

Disease stability

A. The patient's heart failure is stable (next visit within a month or later): → the patient is transferred to a central call center + tele-monitoring;

B. The patient's heart failure is unstable (next visit in less than a month): → disease management will be conducted at the heart failure center until the patient's heart failure is stabilized. When stable, the patient will be transferred for further follow-up at the central call center + tele-monitoring;

Appendix-D:

Sf-36 questionnaire + utility assessment

PHQ-9 questionnaire

References:

1) Ware JE, Snow KK, Kosinski M, Gandek B. SF-36 Health Survey. Manual and interpretation Guide. The Health Institute, New England Medical Center. Boston, MA, 1993.
2) Kroenke K, Spitzer RL. The PHQ-9: A new depression diagnostic and severity measure. Psychiatr Ann 2002; 32:509-15.

Appendix-E: Follow up assessment (every 6 months)

I. Demographic information

Patient name: __________ ___________ I.D. Number |_|_|_|_|_|_|_|_|_| -|_|

Months* from randomization: |_|_|

* -Follow-up visits will be conducted every 6 months from randomization

II. Information on hospital admissions (hospital discharge summaries should be transferred to the Gertner Institute)

1. Hospital admission-1 date: |_|_| - |_|_|- |_|_|_|_|

2. Hospital discharge-1 date: |_|_| - |_|_|- |_|_|_|_|

3. Hospital-1 ____________|_|_|_| discharge ward-1 _____________|_|_|

4. Discharge diagnoses for hospital admission-1:

Diagnosis	***ICD-9 code****
**1.**	|_|_|_| . |_|_|
**2.**	|_|_|_| . |_|_|
**3.**	|_|_|_| . |_|_|
**4.**	|_|_|_| . |_|_|
**5.**	|_|_|_| . |_|_|
**6.**	|_|_|_| . |_|_|
**7.**	|_|_|_| . |_|_|
**8.**	|_|_|_| . |_|_|
**9.**	|_|_|_| . |_|_|
**10.**	|_|_|_| . |_|_|
**11.**	|_|_|_| . |_|_|
**12.**	|_|_|_| . |_|_|

**-to be coded at the Gertner Institute*

To be filled at the Gertner Institute: The patient's heart failure during hospital admission-1 was:

a.) The main cause for hospital admission
b.) Exacerbated during hospital admission for other cause

c.)A non-active diagnosis for a hospital admission due to** (provide diagnosis)**:

***___________ ICD-9 code** |_|_|_| . |_|*

<u>Repeat for each hospital admission within the 6-month period</u>

III. Visits to emergency rooms and acute care services

Date	***Referred to****	***Referral **cause***	***Referral results*****
|_|_| - |_|_| - |_|_|_|_| dd mm yyyy	|_|	|_|	|_|
|_|_| - |_|_| - |_|_|_|_| dd mm yyyy	|_|	|_|	|_|
|_|_| - |_|_| - |_|_|_|_| dd mm yyyy	|_|	|_|	|_|
|_|_| - |_|_| - |_|_|_|_| dd mm yyyy	|_|	|_|	|_|
|_|_| - |_|_| - |_|_|_|_| dd mm yyyy	|_|	|_|	|_|
|_|_| - |_|_| - |_|_|_|_| dd mm yyyy	|_|	|_|	|_|

<u>Codes</u>

**-Referred to: 1=acute care service of Maccabi Health Services; 2=hospital emergency room; 3. Other acute care services (MADA)*

***-Referral cause: 1=Exacerbation of heart failure; 2=Other cause, heart failure was an active problem; 3=Other*

****-Referral result: 1=hospital admission; 2=discharge; 3=death*

IV. Clinical assessment

1. Current chronic medical therapy

Drug	*Dose*	*ATC code*
1.	|_|_|_| mg/gr/units X |_| /day	|_|_|_|_|_|
2.	|_|_|_| mg/gr/units X |_| /day	|_|_|_|_|_|
3.	|_|_|_| mg/gr/units X |_| /day	|_|_|_|_|_|
4.	|_|_|_| mg/gr/units X |_| /day	|_|_|_|_|_|
5.	|_|_|_| mg/gr/units X |_| /day	|_|_|_|_|_|
6.	|_|_|_| mg/gr/units X |_| /day	|_|_|_|_|_|
7.	|_|_|_| mg/gr/units X |_| /day	|_|_|_|_|_|
8.	|_|_|_| mg/gr/units X |_| /day	|_|_|_|_|_|
9.	|_|_|_| mg/gr/units X |_| /day	|_|_|_|_|_|
10.	|_|_|_| mg/gr/units X |_| /day	|_|_|_|_|_|

2. Smoking

I. Does the patient smoke cigarettes?

1. Yes
2. No

II. If positive, how many cigarettes per day, on average? |_|_|_| cigarettes; if less than 1/d write '00'.

III. Did the patient try to quit smoking?

1. Yes
2. No (move to question 3)

IV. Did he/she use the following quitting methods? (allow multiple answers)

1. Nicotine substitutes
2. Buproprion ("Zyban")
3. Smoking-cessation group sessions
4. Other, please specify:____________________ |_|_|

3. Does the patient use the home tele-monitoring equipment?

   a. Yes

   b. No

4. What is the monitoring frequency?

   a. |_|_| times/week

   b. If less than one a week, write |_|_| times/month. If less than 0nce-a-month, write '00'

5. Why did the patient stop using the tele-monitoring equipment?

   a. Following the doctor/nurse order

   b. Due to technical problems

   c. Forgetfulness

   d. Because it is a nuisance

   e. Other, please specify: ___________________|_|_|

V. Follow-up measurements:

1. Weight |_|_|_|.|_| kg

2. Height |_|_|_|.|_| cm

3. Waist circumference |_|_|_|.|_| cm

4. Blood pressure* |_|_|_|/|_|_|_| mmHg

**-Should be measured while the patient is sitting, after at least 5 minutes' rest, while the patient's arm rests on a horizontal surface at the level of the heart. The cuff should match the patient's arm circumference.*

5. Pulse rate* |_|_|_| min

*-Pulse should be measured at rest, after blood pressure measurement

6. Pulse regularity: Regular/ Irregular

VI. Blood tests (to be obtained every six months unless otherwise specified)

<table>
<tr><th>test</th><th>Date (mm/yyyy)</th><th>results</th></tr>
<tr><td>Haemoglobin</td><td>|_|_|/|_|_|_|_|</td><td>|_|_|.|_| gr/dL</td></tr>
<tr><td>Creatinine</td><td>|_|_|/|_|_|_|_|</td><td>|_|.|_| mg/dL</td></tr>
<tr><td>Urea</td><td>|_|_|/|_|_|_|_|</td><td>|_|_| mg/dL</td></tr>
<tr><td>Uric acid</td><td>|_|_|/|_|_|_|_|</td><td>|_|_| mg/dL</td></tr>
<tr><td>Na^+</td><td>|_|_|/|_|_|_|_|</td><td>|_|_|_| meq/L</td></tr>
<tr><td>K^+</td><td>|_|_|/|_|_|_|_|</td><td>|_|_|_| meq/L</td></tr>
<tr><td>Albumin</td><td>|_|_|/|_|_|_|_|</td><td>|_|.|_| mg/dL</td></tr>
<tr><td>Glucose</td><td>|_|_|/|_|_|_|_|</td><td>|_|_|_| mg/dL</td></tr>
<tr><td>HbA1c*</td><td>|_|_|/|_|_|_|_|</td><td>|_|_|.|_|%</td></tr>
<tr><td>Total-cholesterol**</td><td>|_|_|/|_|_|_|_|</td><td>|_|_|_| mg/dL</td></tr>
<tr><td>LDL-cholesterol**</td><td>|_|_|/|_|_|_|_|</td><td>|_|_|_| mg/dL</td></tr>
<tr><td>HDL-cholesterol**</td><td>|_|_|/|_|_|_|_|</td><td>|_|_|_| mg/dL</td></tr>
<tr><td>Triglycerides**</td><td>|_|_|/|_|_|_|_|</td><td>|_|_|_| mg/dL</td></tr>
<tr><td>FT4**</td><td>|_|_|/|_|_|_|_|</td><td>|_|.|_| mcg/dL</td></tr>
<tr><td>TSH**</td><td>|_|_|/|_|_|_|_|</td><td>|_|_| .|_| mU/mL</td></tr>
<tr><td>Vit B12**</td><td>|_|_|/|_|_|_|_|</td><td>|_|_|_|_| pg/mL</td></tr>
<tr><td>Folic Acid**</td><td>|_|_|/|_|_|_|_|</td><td>|_|_|.|_| ng/mL</td></tr>
<tr><td>Iron**</td><td>|_|_|/|_|_|_|_|</td><td>|_|_|_| mcg/dL</td></tr>
</table>

-For diabetic patients only

*****-Once a year***

Six minute walk test: |_|_|_| meter. (Write '000' if not tested; specify the reason for the missing measurement: ________________________|_|_|)

for the missing measurement: ______________________|__|__|)

Resting ECG:

Date of test: |__|__| - |__|__| - |__|__|__|__|

Findings:

1. QRS width |__|__| mm

**Findings** *(mark √ where appropriate)*	**No**	**Yes**
2. LBBB	|_|	|_|
3. RBBB	|_|	|_|
4. Atrial fibrillation	|_|	|_|
5. Pathological Q waves that indicate old MI	|_|	|_|

Was the patient given an implantable device within the last 6 months?

a. **VVI** pacemaker;

b. Atrial-ventricular pacemaker; **DDD**

c. Biventricular pacemaker; **CRT**

d. Biventricular pacemaker and defibrillator (**CRTD**)

e. Defibrillator without pacing (**ICD**)

f. No defibrillator or pacemaker

VII. Clinical evaluation summary and assessment of heart failure severity:

1. Heart failure stage* (mark the appropriate answer)

    C. Structural heart disease with prior or current symptoms of HF

    D. Refractory HF requiring specialized interventions

    *-Source: Hunt SA, Abraham WT, Chin MH, et al. American College of Cardiology; American Heart Association Task Force on Practice Guidelines; American College of Chest Physicians; International Society for Heart and Lung Transplantation; Heart Rhythm Society. ACC/AHA 2005 Guideline Update for the Diagnosis and Management of Chronic Heart Failure in the Adult: a report of the American College of Cardiology/American Heart Association Task Force on Practice Guidelines (Writing Committee to Update the 2001 Guidelines for the Evaluation and Management of Heart Failure): developed in collaboration with the American College of Chest Physicians and the International Society for Heart and Lung Transplantation: endorsed by the Heart Rhythm Society. Circulation, 2005;112(12):e154-235.

2. NYHA functional classification* (mark the appropriate answer)

    I. No limitations of physical activity. No heart failure symptoms;

    IV. Mild limitation of physical activity. Heart failure symptoms with significant exertion; comfortable at rest or with mild activity;

    V. Marked limitation of physical activity. Heart failure symptoms with mild exertion; only comfortable at rest;

    IV. Discomfort with any activity. Heart failure symptoms occur at rest;

*-Source: The Criteria Committee of the New York Heart Association. Diseases of the Heart and Blood Vessels: Nomenclature and Criteria for Diagnosis. 6th ed. Boston, Mass: Little Brown; 1964.

VIII. An update of recommended medical treatment (treatment plan, to be delivered to the primary practitioner):

Drug	*Dose*	*ATC code*
**1.**	|_|_|_| mg/gr/units X |_| /day	|_|_|_|_|_|
**2.**	|_|_|_| mg/gr/units X |_| /day	|_|_|_|_|_|
**3.**	|_|_|_| mg/gr/units X |_| /day	|_|_|_|_|_|
**4.**	|_|_|_| mg/gr/units X |_| /day	|_|_|_|_|_|
**5.**	|_|_|_| mg/gr/units X |_| /day	|_|_|_|_|_|
**6.**	|_|_|_| mg/gr/units X |_| /day	|_|_|_|_|_|
**7.**	|_|_|_| mg/gr/units X |_| /day	|_|_|_|_|_|
**8.**	|_|_|_| mg/gr/units X |_| /day	|_|_|_|_|_|
**9.**	|_|_|_| mg/gr/units X |_| /day	|_|_|_|_|_|
**10.**	|_|_|_| mg/gr/units X |_| /day	|_|_|_|_|_|
**11.**	|_|_|_| mg/gr/units X |_| /day	|_|_|_|_|_|

Effect of Disease Management on Hospital Admissions and Mortality in Ambulatory Patients with Heart Failure: A Randomized Controlled Trial.

Summary of protocol amendments:

1ST AMENDMENT:

Change in inclusion criteria for patients with heart failure recruited from the community. The change was made 6 months after the beginning of the trial (on February, 18th, 2008).

The inclusion criteria for patients recruited from the community in the 1st version of the protocol were:

Inclusion criteria for patients who have not been hospitalized for heart failure within the first two months:

1. Age 18 years or older;
2. The patient has relevant echocardiographic evaluation* performed within 3 months prior to the first evaluation visit in the heart failure centre;
3. The patient has heart failure according to the ESC guidelines (ref. 17);
4. The patient meets at least **one** of the following criteria:
    a. Daily furosemide dose greater than 40 mg;
    b. Chronic treatment with two types of diuretic drugs;
    c. NYHA functional class IV;
    d. Six-minute walking distance <300 meter;
    e. Combined left- and right-heart failure;
    f. At least two hospital admissions for heart failure within the past 12 months;

**If the patient had an acute myocardial infarction during the 3 months prior the screening visit, an updated echocardiographic evaluation has to be performed even if less than 3 months elapsed from the prior assessment.*

The inclusion criteria for patients recruited from the community in the 2nd version of the protocol were:

Inclusion criteria for patients who have not been hospitalized for heart failure within the first two months:

1. Age 18 years or older;
2. The patient has echocardiographic evaluation that includes LVEF assessment. A relevant evaluation for the study is:

   a. Evaluation performed within 3 months prior to the first evaluation visit in the heart failure centre;
   b. Evaluation performed after hospital discharge for acute myocardial infarction, in case the event occurred less than 3 months prior the evaluation visit;
3. The patient has heart failure according to the ESC guidelines (ref. 17);
4. The patient has stage C or D heart failure (see ref. below);
5. The patient has NYHA functional classification III-IV at the time of first evaluation visit.

Hunt SA, Abraham WT, Chin MH, et al. ACC/AHA 2005 guideline update for the diagnosis and management of chronic heart failure in the adult. A report of the American College of Cardiology/American Heart Association Task Force on Practice Guidelines (Committee to Update the 2001 Guidelines for the Evaluation and Management of Heart Failure). *J Am Coll Cardiol* 2005;**46:**1116–43.

2ND AMENDMENT:

Initiation of point-of-care BNP assessments at baseline and follow-up visits. The change was initiated in January 1st, 2009, and included all patients in the trial.

The study ended in July, 31st, 2012. Between August 2012 and November 2013 we invested efforts in data cleaning, obtaining missing hospital discharge summaries, and completion of endpoint adjudication. The SAP was created during this period, and was finalized before data analysis. The study protocol contains a section of a preliminary statistical analysis plan.

STATISTICAL ANALYSIS PLAN
for Disease Management trial of Heart Failure Disease

Final version – 4/11/2013

Written by:

Havi Murad, Ofra Kalter-Leibovici, Michal Benderly, Laurence Freedman

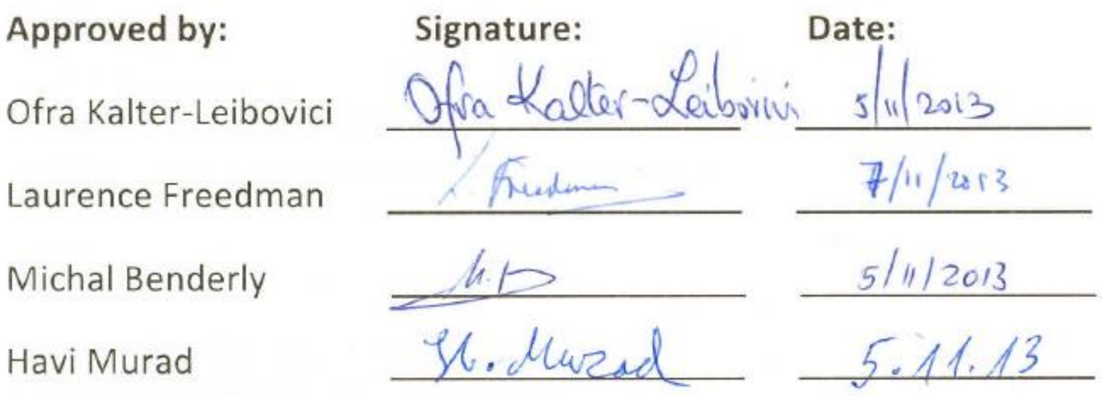

Approved by:	Signature:	Date:
Ofra Kalter-Leibovici	Ofra Kalter-Leibovici	5/11/2013
Laurence Freedman	[illegible]	7/11/2013
Michal Benderly	[illegible]	5/11/2013
Havi Murad	H. Murad	5.11.13

1 SYNOPSIS OF THE STUDY PROTOCOL

1.1 Aim

To study the efficacy of a nurse-led disease management program in community-dwelling patients with moderate-to-severe chronic heart failure (HF).

1.2 Study Design

Open randomized controlled trial, with a parallel-group design and an active control arm.

1.3 Eligibility

- Adult men and women (age: >18 years) with HF who are ensured by Maccabi Health Services in Israel, recruited either <2 months after hospital admission for acute presentation of the disease, or from the community
- Disease severity, assessed by New York Heart Association (NYHA) functional stage:
    - II-IV for patients recruited after hospital admission
    - III-IV for patients recruited from the community
- Heart failure diagnosed according to European Society of Cardiology (ESC) guidelines

1.4 Exclusion Criteria

- Other severe co-morbidity (e.g. end-stage renal failure, severe chronic obstructive pulmonary disease, metastatic cancer)
- Substance abuse (alcohol, illegal drugs)
- People who lack a permanent address or telephone line
- People with significant cognitive impairment / mental disease
- People with severely impaired functional capacity unrelated to heart failure (e.g. bedridden, paraplegic)
- People who are enrolled in another clinical trial
- People who are unwilling to provide a written informed consent

1.5 Randomization

Conducted within 10-patients' blocks and stratified by heart failure center, with 1:1 ratio between the two study arms, maintaining allocation concealment.

1.6 Intervention

1.6.1 Disease Management Study Arm:

a. Patients assigned to this study arm are managed by a multidisciplinary team at designated heart failure centers (n=9) dispersed countrywide. The team includes nurse specialists, cardiologists, social workers and dieticians. The frequency of follow-up visits will be determined according to clinical needs, but no less than once every 6 months.

b. Between visits to the heart failure centers, patients were assigned to a nurse disease manager working at a call center, who was in regular contact with the patients, using either telephone calls or computer video-contacts. These sessions were used to receive updated information on patient's signs and symptoms, to modify medical treatment using designated protocols, to promote patient's lifestyle and medication adherence, to coordinate patient's care, and to provide help in case of acute symptoms.
c. Patients were provided with home tele-monitoring equipment for home blood pressure, heart rate and weight monitoring. The patients' data were automatically entered into the electronic patient record and alerts were produced in case of outlier parameters.

1.6.2 Usual Care Study Arm

Patients assigned to this study arm continued follow-up provided by their family physician and consultant cardiologist. This is the type of care provided to community-dwelling patients with HF by Maccabi Health Services.

1.7 Primary Outcome

First hospital admission for acute exacerbation of heart failure, or death from any cause

1.8 Secondary Outcomes

I. Total number of hospital days and total number of hospital admissions due to HF
II. All-cause mortality
III. Total number of all hospital days and total number of all hospital admissions (for any cause)
IV. Functional capacity, assessed by:
  a. 6-minute walk-test
  b. NYHA stage
V. Health-related quality of life (using the SF-36 questionnaire)
VI. Depression symptomatology (assessed by the PHQ-9 questionnaire)
VII. Change in BNP levels (in a subset of patients)
VIII. Adherence to medical therapy recommended by ESC clinical guideline for patients with chronic heart failure

1.9 Baseline Assessment and Follow-up Visits at the Heart Failure Centers

Baseline evaluation includes a cardiologist-completed questionnaire on eligibility criteria, medical conditions underlying heart disease; cardiovascular risk factors; and other clinical information related to the patients' heart failure (e.g. presence of arrhythmia, implanted pacemaker or ICD).

I. Nurse-completed questionnaire on chronic medical therapy.

II. Nurse-supervised 6-minute walk test.
III. PHQ-9 and SF-36 questionnaires completed by the patient.
IV. BNP on-site assessment (starting from 2009).

1.10 Assessment during Follow-up Visits (every 6 months)

Assessment during follow-up visits included all of the baseline evaluation components described above, except for the physician's questionnaire.

Baseline and follow-up assessment visits were conducted for all randomized patients.

1.11 Study Dates

First patient recruited: August, 2007

Last patient recruited: June, 2011

Total number of patients randomized: 1,361 (one not included in the analysis because informed consent was not provided (protocol violation))

Minimum duration of follow-up: 1 year

Maximal follow-up period: 5 years.

2 HANDLING OF MISSING DATA

Missing covariate data will be handled as far as possible by the method of multiple imputation (Little and Rubin, 2002).

3 SUBJECT WITHDRAWALS DUE TO EARLY TERMINATION

Subjects will be censored at the time of last available information, if the endpoint of interest or any competing risk endpoints have not yet occurred.

4 CLINICAL ENDPOINTS

4.1 Primary Efficacy Endpoint

The primary endpoint of this study is the combined outcome (mortality from any reason or first hospitalization due to heart failure, whichever happens first).

4.2 Secondary Efficacy Endpoints (events are counted until 31/7/12, ignoring early termination)

The arms will be compared with regard to:

1. Mortality from any reason
2. Hospitalization due to HF
3. Total days of hospitalization and total number of hospital admissions due to HF
4. Total days of hospitalization and total number of hospital admissions due to acute exacerbation of HF
5. Total days of hospitalization and total number of hospital admissions due to HF complications(including HF treatment adverse effects)
6. Total days of hospitalization and number of hospital admissions for any cause
7. Functional impairment (NYHA class) at 6 month intervals until the end of follow-up or end of trial
8. 6-minute walk test at 6 month intervals until the end of follow-up or end of trial
9. Quality of life (SF36 and VAS) at 6 month intervals until the end of follow-up or end of trial
10. Depression (PhQ9) adjusted for baseline value (subgroup with baseline value) at 6 month intervals until the end of follow-up or end of trial
11. BNP since 2009 adjusted for baseline value (subgroup with baseline value) at 6 month intervals until the end of follow-up or end of trial
12. Adherence to guidelines for recommended medical treatment

13. Total cost for health care provider to all health care services supplied to the disease management program (not including cost of disease management manpower and infrastructure designated for the study disease program)
14. Total cost of disease management at 6 month intervals until the end of follow-up or end of trial

5 SIGNIFICANCE LEVELS AND HANDLING OF TYPE I ERROR

5.1 Interim Analysis

Eight interim analyses were planned to check for safety with regard to combined outcome and mortality and re-hospitalization. The significance was corrected for multiple testing using the one-sided 5% limit of Pocock in the direction of harm from the experimental management program.
These analyses do not materially affect the type I error rate for the final analysis of efficacy that will examine whether the experimental management program reduces risk in relation to the control group (see Section 5.2).

5.2 Final Analysis

The overall experiment-wise significance level for this study is at a two-tailed 5% level.

6 DATA ANALYSIS SETS AND BASELINE AND FOLLOW-UP VARIABLES

All analyses will be conducted on the Intention to Treat (ITT) dataset according to the treatment group allocated by randomization. This means that all subjects will be kept in their assigned treatment groups, even those who were intended to be in the active group, but for some reason did not receive the experimental disease management program and vice versa.

7 EFFICACY ANALYSIS

7.1 Primary analysis of primary endpoint

The analyses to be conducted at the final statistical analysis are described in this Section. The primary endpoint is time to the combined outcome (mortality or hospitalization due to heart failure, whichever happens first). The Cox regression model will be used to compare the treatment groups with respect to this outcome, and the comparison will be adjusted for the following baseline factors: sex, age (5 categories: <50, 50-59, 60-69, 70-79, 80+), center, and functional impairment (NYHA class) at baseline.

7.2 Secondary analyses

7.2.1 Secondary Analyses of the Primary Endpoint

All analyses will be adjusted for the same baseline factors as mentioned in 7.1 plus others that are unbalanced between the treatment groups, i.e are significantly different at the 10% significance level when entered separately. The additional baseline factors that will be considered are:

A. 6-minute walk test
B. Recruited after hospitalization or from the community
C. Year of recruitment
D. Underlying cause of heart failure: ischemic/non-ischemic
E. Type of heart failure (left ventricular ejection fraction (LVEF) %): preserved (LVEF (%)≥50%)/ non-preserved (LVEF(%)<50%.
F. Co-morbidity: diabetic/ non-diabetic
G. Adherence to HF medication

1. The principal statistical analysis mentioned in 7.1 with additional adjustment.
2. Cox regression for the outcome mortality from any cause.
3. Cox regression with the outcome re-hospitalization due to heart failure.

For each of these outcome models, the following interactions will be tested:

i. Between arm and year of recruitment
ii. Between arm and center
iii. Between arm and recruitment after hospitalization or from the community
iv. Between arm and underlying cause of HF (ischemic/ non-ischemic)
v. Between arm and type of HF (preserved (LVEF≥50%)/ non-preserved (LVEF<50%)
vi. Between arm and co-morbidity (diabetic/ non-diabetic)
vii. For outcomes (1)-(3) above, we will investigate the interaction between treatment arm and time from entry to the trial. This is equivalent to testing the assumption

that the hazards in the two treatment arms are proportional over time – what is known as the proportional hazard assumption.

7.2.2 Analyses of Secondary Endpoints (events are counted until 31/7/12, ignoring early termination)

Fourteen secondary end-points will be analyzed to provide insight into the benefits of the disease management program. The analyses will be conducted on the ITT dataset. All the analyses will be adjusted for the following baseline factors:

A. Sex
B. Age (in five groups, as above)
C. Center
D. Functional impairment (NYHA) at baseline

1. Cox regression with mortality due to any cause.
2. Cox regression with re- hospitalization due to HF
3. Regression with total days of hospitalization and total number of hospital admissions due to HF
4. Regression with total days of hospitalization and total number of hospital admissions due to acute exacerbation
5. Regression with total days of hospitalization and total number of hospital admissions due to HF complications (including HF treatment adverse effects)
6. Regression with total days of hospitalization and number of hospital admissions for any cause
7. Functional impairment (NYHA class) at 6 month intervals
8. 6-minute walk test at 6 month intervals (in whole meters including zeros, probably will be categorized).

The 8th and 9th secondary endpoints are ordered outcomes. Therefore the proportional odds model using the GEE method implemented in the Genmod procedure (SAS 9.2), with repeated measurements, will be fitted. The proportional odds assumption will be tested and if violated a partial proportional odds model will be fitted.

9. Quality of life
   SF36 at 6 month intervals – (score in 2 major domains (physical, mental)) – the GEE method implemented in the Genmod procedure (SAS 9.2), with repeated statement, will be fitted. If the data appear continuous then we will use GEE for a continuous outcome; if there are clumps of measurements (at 0 or 100 say), then we will categorize the data and use the proportional odds model version of GEE.
10. Regression model with repeated measures for Depression (PhQ9) at 6 month intervals adjusted for baseline value (subgroup with baseline value)
11. Regression model with repeated measures for BNP since 2009 at 6 month intervals, adjusted for baseline value (subgroup with baseline value).
12. Adherence to guidelines for recommended medical treatment. Two types of medical treatments will be considered: ACE-I/ARB's and Beta-blockers. We will examine adherence to each individual type and to their combination. Adherence to their combination will be scored 0,1 or 2 for patients who do not take any of the two types, patients taking only one type and patients taking both types of medicine, respectively. The two arms will be compared at 6 month intervals for their adherence to medical treatment using the proportional odds model or the partial proportional odds model using the Genmod procedure (SAS 9.2) with repeated statement.

13. A separate analysis will be conducted on the total cost for health care provider to all health care services supplied to the disease management program (not including cost of disease management manpower and infrastructure designated for the study disease program).
14. Regression model with repeated measures for total cost of disease management at 6 month intervals.
